# Preclinical Comparison of [^111^In]In- and [^225^Ac]Ac-DOTA-Trastuzumab IgG, F(ab′)_2_ and Fab for Theranostic SPECT/CT Imaging and α-Particle Radioimmunotherapy of HER2-Positive Human Breast Cancer

**DOI:** 10.1021/acs.molpharmaceut.4c01071

**Published:** 2024-12-12

**Authors:** Misaki Kondo, Zhongli Cai, Conrad Chan, Madeline K. Brown, Raymond M. Reilly

**Affiliations:** † Department of Pharmaceutical Sciences, University of Toronto, Toronto, ON M5S 3M2, Canada; ‡ Department of Pharmaceutical Sciences, Leslie Dan Faculty of Pharmacy, University of Toronto, 144 College Street, Toronto, ON M5S 3M2, Canada; § Department of Medical Imaging, Temerty Faculty of Medicine, University of Toronto, 263 McCaul St Fourth Floor, Toronto, ON M5S 1A8, Canada; ∥ Joint Department of Medical Imaging and Princess Margaret Cancer Centre, University Health Network, 610 University Ave, Toronto, Ontario M5G 2C1, Canada

**Keywords:** radioimmunotherapy, α-particles, trastuzumab, ^225^Ac, ^111^In, HER2, theranostic, SPECT/CT

## Abstract

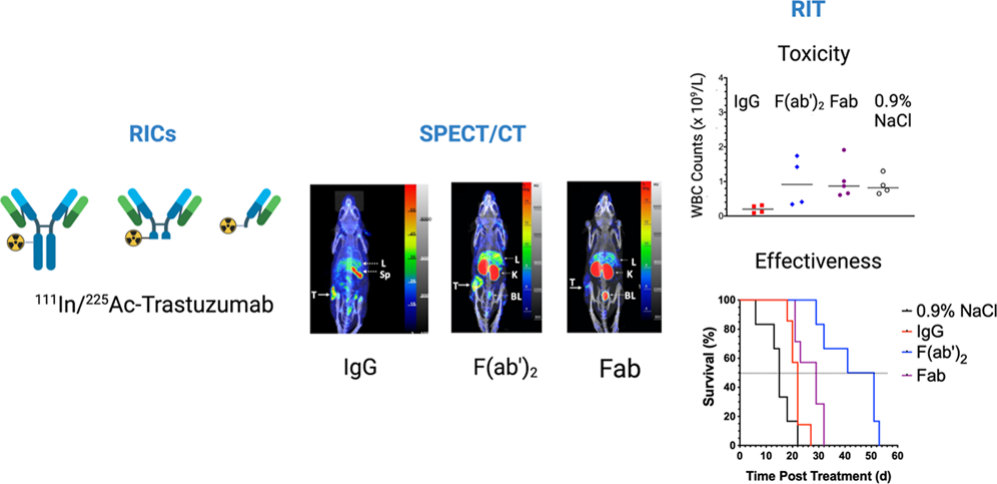

Radioimmunotherapy (RIT) with α-particle-emitting, ^225^Ac complexed to trastuzumab may offer an alternative treatment for patients who progress on HER2-targeted therapies. Moreover, RIT with [^225^Ac]Ac-DOTA-trastuzumab could be combined with SPECT/CT imaging with [^111^In]In-DOTA-trastuzumab in a theranostic approach. In this study, we compared DOTA-conjugated trastuzumab IgG, F(ab')_2_ or Fab complexed to ^111^In or ^225^Ac for SPECT/CT imaging and α-particle RIT of subcutaneous (s.c.) HER2-positive 164/8-1B/H2N.luc^+^ human BC tumors in NRG mice. SPECT/CT imaging and tumor and normal tissue uptake were compared in NRG or NOD-SCID mice coinjected i.v. with [^111^In]In-DOTA-trastuzumab IgG, F(ab')_2_ or Fab and [^225^Ac]Ac-DOTA-trastuzumab IgG, F(ab')_2_ or Fab. Radiation absorbed doses in the tumor and normal organs for [^225^Ac]Ac-DOTA-trastuzumab IgG, F(ab')_2_ or Fab were estimated based on the biodistribution of the [^111^In]In-DOTA-trastuzumab IgG, F(ab')_2_ or Fab. Normal tissue toxicity was assessed by hematology and blood biochemistry analyses and monitoring body weight in NRG mice injected i.v. with 2 and 4 kBq of [^225^Ac]Ac-DOTA-trastuzumab IgG, F(ab')_2_ or Fab separated by 8 d. RIT studies were performed in NRG mice with s.c. 164/8-1B/H2N.luc^+^ tumors injected i.v. with 2 kBq and 4 kBq of [^225^Ac]Ac-DOTA-trastuzumab IgG, F(ab')_2_ or Fab separated by 8 d or irrelevant [^225^Ac]Ac-DOTA-IgG_1_, two doses of unlabeled trastuzumab IgG or 0.9% NaCl. A tumor growth index (TGI) was plotted vs time (d) and Kaplan–Meier median survival estimated. [^111^In]In-DOTA-trastuzumab IgG or F(ab')_2_ exhibited 4.1-fold and 3.3-fold significantly greater tumor uptake at 2 d postinjection (p.i.) than Fab at 24 h p.i. However, spleen uptake at 2 d p.i. for [^111^In]In-DOTA-trastuzumab IgG was 3.3-fold significantly higher than F(ab')_2_ and 13.2-fold higher than Fab at 24 h p.i. [^111^In]In-DOTA-trastuzumab F(ab')_2_ and Fab exhibited higher kidney uptake than IgG. Tumors were imaged by SPECT/CT with [^111^In]In-DOTA-trastuzumab IgG and F(ab')_2_ but were not well-visualized with [^111^In]In-DOTA-trastuzumab Fab. The absorbed dose in the tumor was 2.2-fold greater for [^225^Ac]Ac-DOTA-trastuzumab F(ab')_2_ than IgG and 3.4-fold greater than Fab. Hematological toxicity was observed for [^225^Ac]Ac-DOTA-trastuzumab IgG but not for [^225^Ac]Ac-DOTA-trastuzumab F(ab')_2_ or Fab. No kidney or liver toxicity or decreased body weight was observed for any RIT agent. Tumor growth was significantly inhibited by [^225^Ac]Ac-DOTA-trastuzumab IgG, F(ab')_2_ or Fab but [^225^Ac]Ac-DOTA-trastuzumab F(ab')_2_ was most effective for increasing median survival (46 d vs 22 d for IgG and 29 d for Fab). We conclude that [^111^In]In- and [^225^Ac]Ac-DOTA-trastuzumab F(ab')_2_ exhibited superior properties for theranostic imaging and α-particle RIT of HER2-positive human BC xenografts in NRG mice.

## Introduction

Approximately 15–20% of breast cancers (BC) are classified as human epidermal growth factor receptor-2 (HER2)-positive.^1^ HER2-positive BC was historically associated with a poor prognosis.^2^ However, patient outcomes have improved over recent years due to the introduction of HER2-targeted therapies such as trastuzumab (Herceptin, Roche) and pertuzumab (Perjeta, Roche).^3^ Nonetheless, resistance to HER2-targeted therapies remains a challenge. Antibody-drug conjugates (ADC) such as trastuzumab-emtansine (Kadcyla, Roche) and trastuzumab-deruxtecan (Enhertu, AstraZeneca) can overcome resistance to HER2-targeted therapies since their cytotoxic mechanism of action makes them highly potent, addressing low or heterogeneous HER2 expression.^3^ Trastuzumab-deruxtecan has proven effective for treating BC resistant to trastuzumab^4^ and retains activity against tumors with low HER2.^5^ However, resistance to ADCs develops and alternative treatments with cytotoxic mechanisms of action are needed.^6^

There has been a renaissance in targeted radiotherapies for cancer due to their success in treating neuroendocrine malignancies in patients with β-particle-emitting [^177^Lu]Lu-DOTATATE (Lutathera, Novartis)^7^ and metastatic prostate cancer with [^177^Lu]Lu-PSMA-617 (Pluvicto, Novartis).^8^ Furthermore, there is growing interest in targeted radiotherapy with more powerful α-particle-emitting radionuclides (e.g ^225^Ac), since treatment of metastatic prostate cancer with [^225^Ac]Ac-PSMA-617 was able to overcome resistance to [^177^Lu]Lu-PSMA-617.^9,10^ The high linear energy transfer of α-particles (LET = 50–230 keV/μm) directly causes lethal DNA double-strand breaks (DSB) in cancer cells, while low LET (0.1–1 keV/μm) β-particles rely on indirect DNA damage mediated by reactive oxygen species (ROS) which is susceptible to tumor hypoxia.^11^ Moreover, α-particles are precise for killing cancer cells due to their short-range (28–100 μm), which limits the “cross-fire” effect on nontargeted normal cells, which is an issue for β-particles.^11 225^Ac (*t*_1/2_ = 10 d) emits high energy (5.0–8.5 MeV) α-particles in its decay through several daughter radionuclides that are themselves α- or β-particle emitters to stable ^209^Pb.^11^

Radioimmunotherapy (RIT) combines monoclonal antibodies that target overexpressed receptors on tumors with α- or β-particle-emitting radionuclides.^12^ However, only two preclinical studies have reported α-particle RIT of HER2-positive BC with ^225^Ac-labeled trastuzumab. Yoshida et al.^13^ reported decreased tumor growth in NOD scid gamma (NSG) mice locally injected into the ducts of the mammary gland with [^225^Ac]Ac-DOTA-trastuzumab IgG (1.1–4.4 kBq) for treatment of SUM225 HER2-positive ductal carcinoma in situ (DCIS).^13^ Howe et al.^14^ reported RIT of HER2-positive BT-474 human BC xenografts in NCR nu/nu mice by intravenous (i.v.) injection of 4.6 or 9.2 kBq of [^225^Ac]Ac-DOTA-trastuzumab IgG or alternatively, [^225^Ac]Ac-DOTA incorporated into a pH-responsive liposome. However, a challenge for RIT is hematological toxicity which is dose-limiting for IgG antibodies (MW ∼ 150 kDa) due to their long circulation time in the blood, which results in perfusion and off-target irradiation of the bone marrow.^12^ However, fractionating the administered activity and/or using smaller antibody fragments such as F(ab')_2_ (MW ∼ 110 kDa) or Fab (MW  ∼ 45 kDa) that are eliminated rapidly from the blood by renal excretion minimizes bone marrow toxicity, although this may increase kidney toxicity.^15^ Further, there is a growing interest in applying the theranostic concept, in which imaging with γ-emitting analogues of radiotherapeutic agents may be used to visualize tumors, assess target expression and predict radiation absorbed doses to tumors and normal organs from subsequent α- or β-particle therapy. Since antibody fragments yield higher tumor/blood and tumor/normal tissue ratios than intact IgG forms which improves tumor imaging, these may also offer advantages in a theranostic application.^16^ We describe here a direct comparison of the tumor and normal tissue biodistribution and tumor imaging characteristics of [^111^In]In-DOTA-trastuzumab F(ab')_2_ or Fab compared to intact IgG administered to NRG mice with subcutaneous (s.c.) HER2-positive 164/8-1B/H2N.luc^+^ human BC xenografts. We further compare the radiation absorbed doses in the tumor and normal organs and the effectiveness and normal tissue toxicity of [^225^Ac]Ac-DOTA-trastuzumab IgG, F(ab')_2_ or Fab administered in two fractionated amounts for α-particle RIT of 164/8-1B/H2N.luc^+^ tumors in NRG mice. Our aim was to identify an optimal theranostic pair that could be studied in the future for tumor imaging and α-particle RIT of HER2-positive BC to improve patient outcome, particularly with the emergence of resistance to current standard-of-care therapies.

## Materials and Methods

### Cell Culture and Tumor Xenograft Mouse Model

SK-BR-3 human BC cells (1.3 × 10^6^ HER2/cell)^17^ were purchased from the American Type Culture Collection (ATCC; Manassas, VA). Human 164/8-1B/H2N.luc^+^ BC cells derived from MDA-MB-231 cells transfected with the luciferase gene and HER2 gene^18^ were donated by Dr. Robert S. Kerbel, Sunnybrook Health Sciences Centre, Toronto, Canada. SK-BR-3 and 164/8-1B/H2N.luc^+^ cells were cultured in RPMI 1640 medium (Sigma-Aldrich, St. Louis, MO) supplemented with 10% fetal bovine serum (Gibco; Thermo Fisher Scientific, Waltham, MA) at 5% CO_2_/37 °C. Human BC xenografts were established on the flank in 5–9 week old female NOD-*Rag1*^*null*^*IL2rg*^*null*^ (NRG) or NOD.CB17-*Prkdc*^*scid*^/J (NOD SCID) mice by subcutaneous (s.c.) inoculation of 5 × 10^5^ to 5 × 10^7^ 164/8-1B/H2N.luc^+^ cells mixed with Matrigel (BD Biosciences, Franklin Lakes, NJ) at 1:1 ratio in 200 μL of serum-free media. All animal studies were performed under a protocol (AUP 6509.3) approved by the Animal Care Committee at the University Health Network following Canadian Council on Animal Care (CCAC) guidelines.

### Preparation and Characterization of Trastuzumab F(ab')_2_ and Fab

F(ab')_2_ were prepared by proteolysis of trastuzumab IgG with immobilized pepsin. Briefly, trastuzumab IgG (Herceptin, Roche, Mississauga, ON) was first buffer-exchanged into 20 mM NaOAc buffer, pH 4.5 by transferring 10 mg (0.5 mL) of IgG to an Amicon ultracentrifugal device [30 kDa molecular weight cutoff (MWCO), Amicon] and diluting with 3.5 mL of 20 mM NaOAc buffer, pH 4.5. The device was centrifuged at 7000*g* for 5 min. The retentate was rediluted in 3 mL of 20 mM NaOAc buffer, pH 4.5 and the device centrifuged again, repeated 5 times. The device was subsequently inverted and centrifuged at 330*g* for 2 min to recover the buffer-exchanged IgG. The IgG concentration was measured spectrophotometrically (*A*_280_ of a 1 mg/mL solution = 1.47). IgG (6.1 mg/mL) in 20 mM NaOAc buffer, pH 4.5) and was then reacted with immobilized pepsin (ThermoFisher Scientific) at a ratio of 0.25 mg of pepsin resin (0.25 mL slurry) per 4 mg of IgG at 37 °C for 20 h on a nutating mixer (VWR, Mississauga, ON). After incubation, the resin mixture was suspended with ice-cold phosphate buffered saline (PBS), pH 7.4 containing 10 mM Tris-HCl (pH 7.5) and centrifuged at 1000*g* for 5 min and the supernatant collected, repeated twice. The supernatants containing pure trastuzumab F(ab')_2_ were pooled.

Fab were prepared by first buffer-exchanging 10 mg of trastuzumab IgG into 20 mM NaH_2_PO_4_ buffer, pH 7.0 containing 10 mM Na_2_EDTA on an Amicon ultracentrifugal unit (30 kDa MWCO) as described above. Buffer-exchanged IgG was recovered, and the concentration measured spectrophotometrically (*A*_280_ of a 1 mg/mL solution = 1.47). Immediately prior to reaction with IgG, the mass of immobilized papain required to produce Fab (1 mg of papain resin (8 mL slurry) per 50 mg of IgG) was obtained and prepared for reaction by equilibrating with freshly produced 20 mM NaH_2_PO_4_ pH 7.0 buffer containing 10 mM Na_2_EDTA and 80 mM l-cysteine (digestion buffer) at 1:4 v/v and vortexed. The slurry was centrifuged at 200*g* for 5 min and the supernatant removed and discarded, repeated twice. Rinsed immobilized papain was reacted with IgG at a ratio of 1 mg of papain resin (8 mL slurry) per 50 mg of IgG in digestion buffer at a final concentration of 1.38 mg/mL. The reaction tube was purged with N_2_ for 2–3 min and promptly capped. The reaction mixture was then gently mixed for 20 h at 37 °C in an Excella E24 Incubator Shaker (New Brunswick Scientific, Edison, NJ) at 300 rpm. After incubation, 1.5 mL of 10 mM Tris-HCl, pH 7.5 was added and the tube was shaken rigorously. The resin suspension was diluted with 3–4 mL of 100 mM NaHCO_3_ buffer, pH 8.2 and centrifuged at 200*g* for 5 min to collect the supernatant. This was repeated a total of 3 times. The supernatants containing Fab were pooled and filtered through a Millex-GV PDVF 0.22 μm filter to remove residual resin. F(ab')_2_ and Fab were buffer-exchanged into 100 mM NaHCO_3_ buffer, pH 8.2 on an Amicon device (30 kDa MW cutoff) centrifuged at 7000*g* for 5 min.

The recovered F(ab')_2_ and Fab were adjusted to a final concentration of 20–25 mg/mL in 100 mM NaHCO_3_ buffer, pH 8.2. F(ab')_2_ and Fab concentrations were measured spectrophotometrically (*A*_280_ of a 1 mg/mL solution = 1.45 for F(ab')_2_ and 1.40 for Fab). Optimization of proteolytic digestion conditions for producing immunoreactive and pure F(ab')_2_ and Fab are described in the Supporting Information. Trastuzumab F(ab')_2_ and Fab were analyzed for purity and homogeneity by SDS-PAGE under nonreducing and reducing [dithiothreitol (DTT)] conditions on a 7.5% Mini-Protean Tris/glycine mini-gel (BioRad, Mississauga, ON, Canada) with the bands stained with Biosafe Coomassie Blue G-250 (BioRad).

### DOTA Conjugation of Trastuzumab IgG, F(ab')_2_ and Fab

Trastuzumab IgG (15–20 mg/mL in 100 mM NaHCO_3_ buffer) or irrelevant human IgG_1_ (BE0297, Cedarlane Laboratories, Burlington, ON) were reacted with a 30-fold excess of 1,4,7,10-tetraazacyclododecane-1,4,7,10-tetraacetic acid *N*-hydroxysuccinimide ester (NHS-DOTA; Macrocyclics, Dallas, TX) as previously reported^19^ resulting in conjugation of 12.7 ± 1.2 DOTA. Trastuzumab F(ab')_2_ and Fab (15–20 mg/mL in 100 mM NaHCO_3_ buffer, pH 8.2) were reacted with a 20-fold or 40-fold molar excess of NHS-DOTA, respectively, resulting in conjugation of 13.8 ± 1.3 and 12.1 ± 0.5 DOTA. Optimization of DOTA conjugation conditions are described in the Supporting Information. The purity of F(ab')_2_ and Fab and the DOTA-immunoconjugates was determined by sodium dodecyl sulfate polyacrylamide gel electrophoresis (SDS-PAGE) on a 7.5% Mini-Protean Tris/glycine mini-gel (BioRad, Mississauga, ON, Canada).

### [^111^In]In- and [^225^Ac]Ac-DOTA-Trastuzumab IgG, F(ab')_2_ or Fab

DOTA-trastuzumab IgG, F(ab')_2_ or Fab or irrelevant IgG_1_ were buffer-exchanged into 100 mM NH_4_Ac buffer, pH 5.5 and concentrated to 20–30 mg/mL as described in the Supporting Information. Labeling with ^111^In or ^225^Ac was performed as previously reported.^19^ Briefly, DOTA-trastuzumab IgG, F(ab')_2_ or Fab (100 μg in 100 mM NH_4_Ac buffer, pH 5.5) were incubated with [^111^In]InCl_3_ (BWXT, Ottawa, ON, Canada) mixed with an equal volume of 100 mM NH_4_Ac buffer, pH 5.5 at 40 °C for 1 h. Postlabeling purification was not required. DOTA-trastuzumab IgG, F(ab')_2_ or Fab were labeled with ^225^Ac as reported by Maguire et al.^20^ Approximately 37 MBq of dry [^225^Ac]Ac(NO_3_)_3_ (U.S. Department of Energy Isotope Program, Oak Ridge, TN) was dissolved in 10 μL of 0.2 M Optima grade high purity HCl (Thermo Fisher Scientific). ^225^Ac (0.8–1.5 MBq) in 0.2 M HCl was mixed with 25 μL of 2 M tetramethylammonium acetate buffer and 10 μL of l-ascorbic acid (150 mg/mL) then incubated with DOTA-trastuzumab IgG, F(ab')_2_ or Fab (100–250 μg) in 4–10 μL of 100 mM NH_4_Ac buffer, pH 5.5 at 37 °C for 2 h. [^225^Ac]Ac-DOTA-trastuzumab IgG and F(ab')_2_ or Fab were purified on an Amicon device. The final radiochemical purity (RCP) was determined by instant thin layer silica gel chromatography (ITLC-SG; Agilent Technologies, Santa Clara, CA) developed in 0.1 M Na citrate buffer, pH 5.5. The R_f_ values were 0.0 for [^111^In]In- or [^225^Ac]Ac-DOTA-trastuzumab IgG, F(ab')_2_ or Fab and 1.0 for free ^111^In or ^225^Ac. The specific activity (SA) of [^111^In]In-DOTA-trastuzumab IgG, F(ab')_2_ or Fab were 0.1–0.25 MBq/μg (1.5–3.8 × 10^16^ Bq/mol), 0.1–0.29 MBq/μg (1.0–2.96 × 10^16^ Bq/mol) and 0.1–0.29 MBq/μg (0.52–1.5 × 10^16^ Bq/mol), respectively. The SA of [^225^Ac]Ac-DOTA-trastuzumab IgG, F(ab')_2_ or Fab were 1.85–5.6 kBq/μg (2.8–8.4 × 10^14^ Bq/mol), 2.9–5.2 kBq/μg (3.0–5.3 × 10^14^ Bq/mol) and 3.0–7.7 kBq/μg (1.6–4.0 × 10^14^ Bq/mol), respectively.

### HER2 Immunoreactivity

The HER2 immunoreactivity of [^111^In]In-DOTA-trastuzumab IgG, F(ab')_2_ or Fab conjugated at different levels with DOTA was evaluated in a single concentration binding assay as described in the Supporting Information. The HER2 binding properties of optimized radioimmunoconjugates (RICs) were then determined in a direct (saturation) radioligand binding assay using HER2-positive SK-BR-3 human BC cells (4 × 10^5^ HER2/cell) as previously reported.^19^ Briefly, the dissociation constant (*K*_D_) and maximum number of binding sites per cell (*B*_max_) for binding of [^111^In]In-DOTA-trastuzumab IgG, F(ab')_2_ or Fab to HER2-positive SKBR-3 cells were measured in a saturation radioligand binding assay. SK-BR-3 cells (1 × 10^6^ cells) were incubated with increasing concentrations (0.073–300 nmol/L) of [^111^In]In-DOTA-trastuzumab IgG, F(ab')_2_ or Fab (0.11 MBq/μg) in 1.5 mL Eppendorf tubes in the absence or presence of a 50-fold molar excess of trastuzumab IgG for 3.5 h at 4 °C to measure total binding (TB) and nonspecific binding (NSB), respectively. The tubes were gently agitated every 30 min. The tubes were then centrifuged at 1300*g* for 5 min on an Eppendorf microcentrifuge to remove unbound activity in the supernatant. The cell pellets were rinsed twice with 500 μL of ice-cold PBS, pH 7.4. The combined supernatants containing unbound activity and cell pellets containing cell-bound activity (TB or NSB) were measured in a γ-counter. NSB was subtracted from TB to obtain specific binding (SB). TB, NSB and SB of [^111^In]In-DOTA-trastuzumab F(ab')_2_ or Fab (pmoles) were plotted vs the concentration of free (unbound) radioligands (nmol/L) and the curve was fitted to a one-site-receptor-binding model using Prism Ver. 8.0 software (GraphPad, San Diego, CA) to estimate the *K*_D_ and *B*_max_. The HER2 immunoreactivity of [^225^Ac]Ac-DOTA-trastuzumab IgG, F(ab')_2_ or Fab were not determined as it was assumed that substitution of ^225^Ac for ^111^In would not change HER2 immunoreactivity. This was based in our observation of equivalent cell–surface binding and intracellular distribution of [^225^Ac]Ac-DOTA-trastuzumab IgG and [^111^In]In-DOTA-trastuzumab IgG measured in SK-BR-3 cells (Figure S3). Furthermore, Borchardt et al.^21^ reported similar binding of [^111^In]In-DOTA-trastuzumab IgG and [^225^Ac]Ac-DOTA-trastuzumab IgG in vitro to HER2-positive SK-OV-3 cells. Radiolysis due to α-particle emissions by ^225^Ac may decrease immunoreactivity but ascorbic acid was included in the ^225^Ac-labeling method as a free radical scavenger.^20^

### Biodistribution and SPECT/CT Imaging

The tumor and normal tissue biodistribution of [^111^In]In-DOTA-trastuzumab IgG, F(ab')_2_ or Fab mixed with [^225^Ac]Ac-DOTA-trastuzumab IgG, F(ab')_2_ or Fab were determined in NRG or NOD/SCID mice with s.c. HER2-positive 164/8-1B/H2N.luc^+^ human BC xenografts. ^111^In was used to trace the uptake of ^225^Ac by γ-counting due to the very low amounts of ^225^Ac-labeled RICs administered (4–6 kBq) and the absence of γ-emissions by ^225^Ac. ^225^Ac may be measured in tissues by γ-counting of the ^221^Fr [Eγ = 218 keV (11.4%)] or ^213^Bi (Eγ = 440 keV (25.9%)] daughters but this may not be accurate if there is not secular equilibrium, which may be the case if there is release and redistribution of these daughters following α-particle decay of ^225^Ac.^22^ Additionally, ^111^In enabled single photon emission computed tomography/computed tomography (SPECT/CT) imaging to visualize tumor and normal tissue uptake. Mice were injected intravenously (i.v.; tail vein) with RICs administered at the same total molar mass (2.63 × 10^–10^ moles). NRG mice were coinjected with 2–22 MBq (3.4–35 μg) of [^111^In]In-DOTA-trastuzumab IgG mixed with 4 kBq (1.4 μg) [^225^Ac]Ac-DOTA-trastuzumab IgG supplemented with DOTA-trastuzumab IgG (4.2–36.6 μg; total mass = 40.0 μg) in 160 μL of 0.9% NaCl. At 1 h, 2 d, 7 and 14 d postinjection (p.i.) groups of mice (n = 4–5) were sacrificed under 2% isoflurane anesthesia in O_2_. The tumor was excised, and blood samples and normal tissues collected, weighed and ^111^In measured in a γ-counter or radioisotope dose calibrator (carcass). Tissue uptake of ^111^In was expressed as percent injected dose/gram (% ID/g). Similarly, NOD/SCID mice (*n* = 4–5) were coinjected i.v. with 1–8.7 MBq (7.7–24.4 μg) of [^111^In]In-DOTA-trastuzumab F(ab')_2_ mixed with 4 kBq (2.5 μg) of [^225^Ac]Ac-DOTA-trastuzumab F(ab')_2_ supplemented with DOTA-trastuzumab F(ab')_2_ (0.01–16.7 μg; total mass = 26.9 μg). Finally, NOD/SCID mice (*n* = 4–5) were coinjected i.v. with 1–5.5 MBq of [^111^In]In-DOTA-trastuzumab Fab mixed with 4 kBq (1.3 μg) of [^225^Ac]Ac-DOTA-trastuzumab Fab supplemented with DOTA-trastuzumab Fab (0.02–3.8 μg; total mass = 13.8 μg). Biodistribution was determined at 2 h, 6 h, 1 and 2 d p.i. for mice receiving [^111^In]In- and [^225^Ac]Ac-DOTA-trastuzumab F(ab')_2_ and at 2, 6, 18 h and 1 d p.i. for [^111^In]In- and [^225^Ac]Ac-DOTA-trastuzumab Fab.

SPECT/CT imaging was performed at 2 d p.i. in NOD/SCID mice with s.c. 164/8-1B/H2N.luc^+^ tumor xenografts injected with 7–7.2 MBq of [^111^In]In-DOTA-trastuzumab F(ab')_2_ mixed with 4 kBq of [^225^Ac]Ac-DOTA-trastuzumab F(ab')_2_ (total mass = 35 μg) or at 18 h p.i. in mice injected with 1–3.7 MBq of ^[111^In]In-DOTA-trastuzumab Fab mixed with 4 kBq of [^225^Ac]Ac-DOTA-trastuzumab Fab (total mass = 13.8 μg). SPECT/CT images were previously reported at 2 d p.i. of 7–8 MBq of [^111^In]In-DOTA-trastuzumab IgG mixed with 4 kBq of [^225^Ac]Ac-DOTA-trastuzumab IgG (total mass = 40 μg) in NRG mice with s.c. 164/8-1B/H2N.luc^+^ tumor xenografts.^19^ For SPECT/CT, mice were anaesthetized using 2% isoflurane in O_2_ and imaged in a prone position on a trimodality NanoScan SPECT/CT/PET system (Mediso, Budapest, Hungary) equipped with 4 NaI (Tl) detectors. SPECT employed a 40 s/frame acquisition time resulting in a scan duration of 45 min. CT images were acquired using parameters of 50 kVp X-rays, 980 μA and 300 msec exposure time, isotropic voxel size of 125 μm and maximum field-of-view with 1:4 binning. Images were reconstructed using TeraTomo 3D Normal Dynamic Range Monte Carlo-based reconstruction protocol with a 128 × 128 reconstruction matrix, with three subsets of data undergoing 48 iterations applied with CT-based attenuation and scatter correction. SPECT and CT images were coregistered with InterView Fusion software (Ver. 3.09; Mediso).

### Tumor and Normal Organ Dosimetry

The absorbed doses in the tumor and normal organs in mice injected i.v. with [^225^Ac]Ac-DOTA-trastuzumab IgG, F(ab')_2_ or Fab were estimated based on the biodistribution (%ID/g) of [^111^In]In-DOTA-trastuzumab IgG, F(ab')_2_ or Fab, assuming that ^111^In- and ^225^Ac-labeled RICs distribute equivalently. Borchardt et al.^21^ reported similar biodistribution of intraperitoneally (i.p.)-injected [^111^In]In-DOTA-trastuzumab IgG and [^225^Ac]Ac-DOTA-trastuzumab IgG in athymic mice with intraperitoneal HER2-positive SK-OV-3 human ovarian cancer xenografts. To estimate dosimetry, at least 3 biodistribution time points selected at one-third, one times and 3 times the effective half-life (*t*_1/2e_) of a radiopharmaceutical are recommended.^23^ Thus, we performed biodistribution studies at four time points that were selected based on the relative elimination rates of [^225^Ac]Ac-DOTA-trastuzumab IgG, F(ab')_2_ or Fab. ^225^Ac uptake (MBq/kg per kBq injected) in the tumor, normal organs and carcass were calculated at 1 h, 2 d, 7 and 14 d p.i. for [^225^Ac]Ac-DOTA-trastuzumab IgG; 2 h, 6 h, 1d and 2 d p.i. for [^225^Ac]Ac-DOTA-trastuzumab F(ab')_2__2_; and 2 h, 6 h, 18 h and 1 d p.i. for [^225^Ac]Ac-DOTA-trastuzumab Fab and plotted vs time p.i. (min). The time-integrated activity (MBq·min/kg) from time zero p.i. to the final measured time point (Ã_0–*t*_) was obtained from the area-under-the-curve (AUC). To estimate the time-integrated activity from the final measured time point (A_*t*_) to infinity (Ã_*t*–∞_), the data points from the maximum. ^225^Ac uptake were fitted to a one-phase decay [*A* = *A*_0_ × exp(−*k* × *t*)] to derive an effective elimination constant (*k*) that included radioactive decay and biological clearance. *A*_*t*_ was divided by this constant, *k* to obtain Ã_*t*–∞_. However, for [^225^Ac]Ac-DOTA-trastuzumab F(ab')_2_ in the tumor and spleen and [^225^Ac]Ac-DOTA-trastuzumab Fab in the tumor, maximum uptake had not yet been reached or appeared to plateau at the last measured time point, thus, Ã_*t*–∞_ were estimated by dividing A_*t*_ by the decay constant of ^225^Ac (4.81 × 10^–5^ min^–1^), assuming further elimination solely by radioactive decay. The total time-integrated activity (Ã_0–∞_) was calculated as the sum of Ã_0–*t*_ and Ã_*t*–∞_. For dose estimates, it was assumed that all ^225^Ac daughters deposit their energy in the same tissue as ^225^Ac and only the energy deposited by α-particles from ^225^Ac and its daughters was considered. Based on the Medical Internal Radiation Dose (MIRD) decay schemes,^24^ the energy deposited by α-particle emission per ^225^Ac decay chain was calculated as 27.99 MeV Bq^−^^1–^s^–1^ or 2.69 × 10^–4^ J MBq^–1^ min^–1^, while only 0.0311 MeV Bq^-^^1–^s^–1^ was deposited by all emitted electrons and photons combined, which was 0.1% of the energy deposited by α-particles. The absorbed dose (Gy/kBq) in each tissue deposited by ^225^Ac was calculated by multiplying Ã_0–∞_ (MBq min kg^–1^ kBq^–1^) by 2.69 × 10^–4^ J MBq^–1^ min^–1^.

### Normal Tissue Toxicity

The normal tissue toxicity of [^225^Ac]Ac-DOTA-trastuzumab IgG, F(ab')_2_ and Fab were assessed in nontumor bearing NRG mice. NRG mice were selected for normal tissue toxicity studies and RIT studies since they do not harbor the *scid* gene mutation which makes NOD/SCID mice sensitive to the DNA-damaging effects of ionizing radiation.^25^ Groups of mice (*n* = 4–5) were injected i.v. (tail vein) with 2 kBq (40 μg) and 4 kBq (40 μg) of [^225^Ac]Ac-DOTA-trastuzumab IgG, 2 kBq (27 μg) and 4 kBq (27 μg) of [^225^Ac]Ac-DOTA-trastuzumab F(ab')_2_, or 2 kBq (14 μg) and 4 kBq (14 μg) of [^225^Ac]Ac-DOTA-trastuzumab Fab in 160 μL of 0.9% NaCl separated by 8 d. Control mice were injected i.v. with 160 μL of 0.9% NaCl separated by 8 d. General health and body weight were monitored for 14 d. Mice were then sacrificed under 2% isoflurane in O_2_ anesthesia. Blood was collected into ethylenediaminetetraacetic acid (EDTA) coated capillary tubes (Sarstedt, Nümbrecht, Germany) and analyzed on a VetScan HM5 (Abaxis, Union City, CA) instrument for hematological analysis. For biochemical analysis, blood was collected in lithium heparin-coated capillary tubes (Sarstedt) and loaded onto VetScanPrep Profile II reagent rotors (Abaxis), then analyzed on a VetScan VS2 Analyzer (Abaxis) for blood alanine aminotransferase (ALT) and creatinine (CRE) levels.

### Radioimmunotherapy

NRG mice with s.c. 164/8-1B/H2N.luc^+^ tumors (*n* = 6–7) were treated by i.v. injection of 2 kBq (40 μg) and 4 kBq (40 μg) of [^225^Ac]Ac-DOTA-trastuzumab IgG, 2 kBq (27 μg) and 4 kBq (27 μg) of [^225^Ac]Ac-DOTA-trastuzumab F(ab')_2_, or 2 kBq (14 μg) and 4 kBq (14 μg) of [^225^Ac]Ac-DOTA-trastuzumab Fab in 160 μL of 0.9% NaCl separated by 8 d. Controls consisted of tumor-bearing mice injected with 2 kBq (40 μg) and 4 kBq (40 μg) of irrelevant [^225^Ac]Ac-DOTA-IgG_1_ in 160 μL of 0.9% NaCl, two doses of unlabeled trastuzumab IgG (40 μg each), or two doses of 0.9% NaCl separated by 8 d. Treatment was commenced when the tumors reached ∼5 mm in diameter (16–17 d postinoculation of 164/8-1B/H2N.luc + cells). Tumor size and body weight were measured every 2–3 d until a humane end point was reached. Tumor size was measured by calipers and tumor volume (*V*; mm^3^) was calculated as *V* = (length × width^2^)/2. The tumor growth index (TGI) was calculated as the tumor volume at each observation time point divided by the volume at the start of treatment. The proportion of surviving mice was plotted vs time since treatment and the median survival (*d*) in each treatment group was compared by Kaplan–Meier analysis.

### Statistical Analyses

Data were expressed as mean ± SD or SEM. Statistical analyses were carried out with Prism Ver. 10.1 (GraphPad) using Welch’s *t*-test or one-way ANOVA (*P* < 0.05). Median survival was compared by the log-rank (Mantel–Cox) test (*P* < 0.05).

## Results

### Characterization of Trastuzumab IgG, F(ab')_2_ or Fab

**Figure 1:**
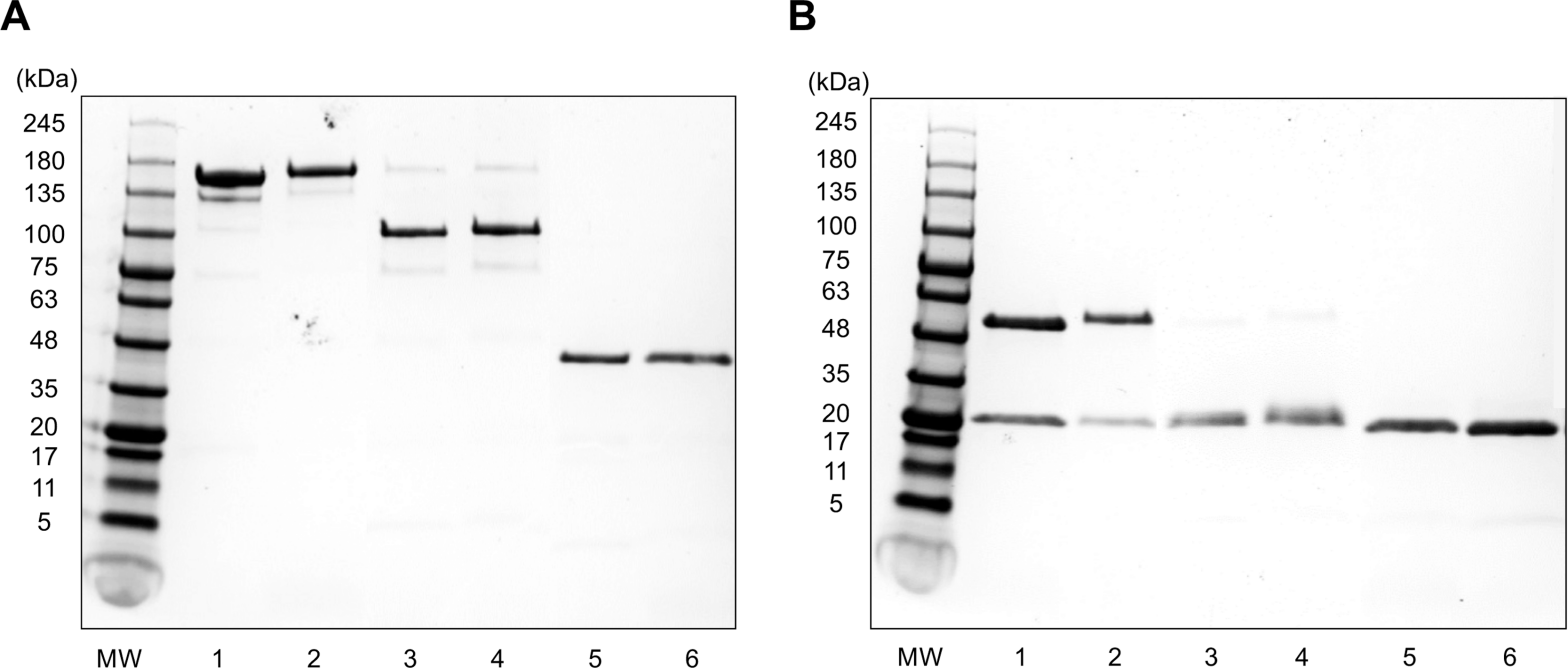
SDS-PAGE of trastuzumab (lane 1), DOTA-trastuzumab (lane 2), trastuzumab F(ab')_2_ (lane 3), DOTA-trastuzumab F(ab')_2_ (lane 4) trastuzumab Fab (lane 5), and DOTA-trastuzumab Fab (lane 6), under nonreducing conditions (A) and reducing conditions (B) on a 4–20% Mini-Protean Tris/glycine mini-gel. Bands were stained with Coomassie Brilliant Blue G-250. MW: Molecular weight markers.

Trastuzumab F(ab')_2_ or Fab were pure as evidenced by migration as a single major band under nonreducing conditions on SDS-PAGE corresponding to the expected MW = 100 or 44 kDa, respectively (Figure 1A). Trastuzumab IgG migrated as a single major band corresponding to MW = 153 kDa. There were some minor bands visible for trastuzumab F(ab′)_2_ at MW = 168 kDa and 77 kDa, but these accounted for <5% of the intensity of all bands. No additional bands were visible for Fab. Under reducing conditions, trastuzumab IgG migrated as two major bands at 52 and 20 kDa, representing the dissociated heavy and light chains (Figure 1B). Trastuzumab F(ab')_2_ or Fab migrated as a single band under reducing conditions, corresponding to the variable and constant regions of the heavy and light chains: *V*_H_–*C*_H_ and *V*_L_–*C*_L_, which have MW = 20 kDa.

### [^111^In]In- and [^225^Ac]Ac-DOTA-Trastuzumab IgG, F(ab')_2_ or Fab

**Figure 2:**
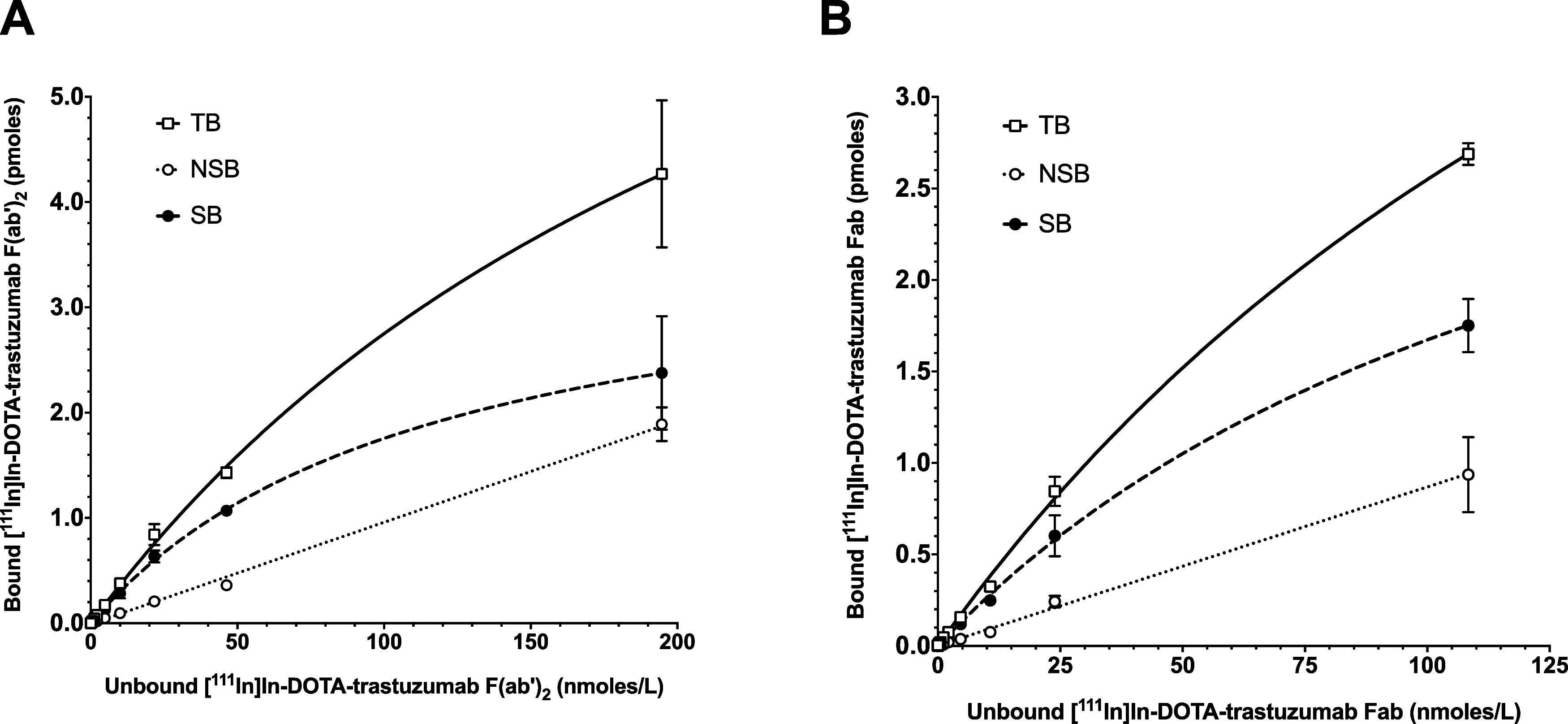
Binding of [^111^In]In-DOTA-trastuzumab F(ab')_2_ (A) and [^111^In]In-DOTA-trastuzumab Fab (B) to HER2-positive SK-BR-3 human BC cells in the absence (total binding; TB) or presence (nonspecific binding; NSB) of a 50-fold molar excess of trastuzumab. Specific binding (SB) was calculated by subtracting NSB from TB.

Trastuzumab IgG was conjugated to 12.7 ± 1.2 DOTA/IgG as previously reported.^19^ Trastuzumab F(ab')_2_ and Fab were conjugated to 13.8 ± 1.3 DOTA and 12.1 ± 0.5 DOTA, respectively. The labeling efficiency (LE) of DOTA-trastuzumab IgG with ^111^In was 93.8 ± 1.6%^19^ and was 96.8 ± 1.0% and 96.9 ± 1.4% for F(ab')_2_ or Fab, respectively. The LE of DOTA-trastuzumab IgG, F(ab')_2_ or Fab with ^225^Ac was 76.4 ± 3.1%,^19^ 62.3 ± 11.8% and 71.0% ± 2.1%, respectively. After postlabeling purification, the RCP of [^225^Ac]Ac-DOTA-trastuzumab IgG, F(ab')_2_ or Fab was 95.9 ± 0.9%,^19^ 92.0 ± 1.5% and 91.0 ± 2.7%, respectively. [^111^In]In-DOTA-trastuzumab F(ab')_2_ and [^111^In]In-DOTA-trastuzumab Fab prepared under optimized conditions exhibited saturable and specific binding to HER2-positive SK-BR-3 cells (Figure 2). The K_D_ and *B*_max_ of [^111^In]In-DOTA-trastuzumab F(ab')_2_ and Fab were 1.2 ± 0.3  × 10^–7^ and 1.4 ± 0.3  × 10^–7^ mol/L, respectively and 2.2 ± 0.3  × 10^6^ and 2.4 ± 0.3  × 10^6^ receptors/cell, respectively. The *K*_D_ and *B*_max_  values of [^111^In]In-DOTA-trastuzumab IgG for binding to SK-BR-3 cells were 1.2 ± 0.3  × 10^–8^ mol/L and 4.2 ± 0.2  × 10^6^ receptors/cell.^19^ The *K*_D_ and *B*_max_ values for [^225^Ac]Ac-DOTA-trastuzumab IgG, F(ab')_2_ or Fab were not determined as it was assumed that substitution of ^111^In for ^225^Ac would not affect HER2 immunoreactivity.

### Biodistribution and SPECT/CT Imaging

**Figure 3:**
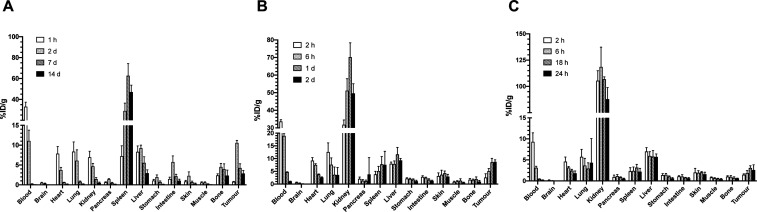
Tumor and normal tissue uptake of (A) [^111^In]In-DOTA-trastuzumab IgG (2–22 MBq) mixed with [^225^Ac]Ac-DOTA-trastuzumab IgG (4 kBq; total mass = 40 μg), (B) [^111^In]In-DOTA-trastuzumab F(ab')_2_ (1–8.7 MBq) mixed with [^225^Ac]Ac-DOTA-trastuzumab F(ab')_2_ (4 kBq; total mass = 26.9 μg), and (C) [^111^In]In-DOTA-trastuzumab Fab (1–5.5 MBq) mixed with [^225^Ac]Ac-DOTA-trastuzumab Fab (4 kBq; total mass = 13.8 μg) in NRG mice (panel A) or NOD/SCID mice (panels B and C) with s.c. HER2-positive 164/8-1B/H2N.luc^+^ human BC tumors. Tissue activity was quantified by γ-counting of ^111^In. Values are the mean ± SD (*n* = 4–5).

In NRG mice with s.c. HER2-positive human 164/8-1B/H2N.luc^+^ tumors injected with [^111^In]In-DOTA-trastuzumab IgG, activity in the blood decreased from 33.1 ± 4.3% ID/g at 1 h p.i. to 11.0 ± 2.7% ID/g, 0.2 ± 0.03% ID/g and 0.06 ± 0.02% ID/g at 2 d, 7 and 14 d p.i., respectively (Figure 3A). Tumor uptake increased from 0.8 ± 0.1% ID/g at 1 h p.i. to 10.6 ± 0.6% ID/g at 2 d p.i., then decreased to 4.2 ± 1.0% ID/g and 2.9 ± 0.7% ID/g at 7 and 14 d p.i., respectively. Spleen exhibited the highest normal tissue uptake, increasing from 7.2 ± 2.6% ID/g at 1 h p.i. to 29.0 ± 7.4% ID/g at 2 d, then reaching a maximum of 62.7 ± 11.5% ID/g at 7 d p.i., before decreasing to 47.1 ± 6.6% ID/g at 14 d p.i. Liver uptake was 8.3 ± 1.5% ID/g at 1 h p.i. and 9.2 ± 0.8% ID/g at 2 d p.i., then decreased to 5.5 ± 1.5% ID/g and 3.0 ± 0.8% ID/g at 7 and 14 d p.i., respectively. Kidney uptake was 6.9 ± 1.5% ID/g at 1 h p.i. and 4.7 ± 0.8% ID/g at 2 d p.i., then decreased to 1.5 ± 0.4% ID/g and 0.5 ± 0.2% ID/g at 7 and 14 d p.i., respectively.

In NOD/SCID mice injected with [^111^In]In-DOTA-trastuzumab F(ab')_2_, blood activity decreased from 33.6 ± 1.0% ID/g at 2 h p.i. to 18.9 ± 0.9% ID/g, 4.7 ± 0.1% ID/g and 1.1 ± 0.1% ID/g at 6 h, 1 and 2 d p.i., respectively (Figure 3B). Tumor uptake increased from 2.6 ± 1.5% ID/g at 2 h p.i., to 5.0 ± 1.0% ID/g, 8.6 ± 1.5% ID/g and 8.7 ± 0.8% ID/g at 6 h, 1 and 2 d p.i., respectively. Tumor uptake at 2 d p.i. was 1.2-fold significantly lower than [^111^In]In-DOTA-trastuzumab IgG (10.6 ± 0.6% ID/g; *P* = 0.004). The kidneys exhibited the highest normal tissue uptake increasing from 31.8 ± 0.9% ID/g at 2 h p.i., to 51.2 ± 6.7% ID/g and 70.2 ± 8.1% ID/g at 6 h and 1 d p.i., respectively, then decreased to 49.7 ± 5.5% ID/g at 2 d p.i. At 2 d p.i., spleen uptake (7.6 ± 5.3% ID/g) was 3.8-fold significantly lower than [^111^In]In-DOTA-trastuzumab IgG (29.0 ± 7.4% ID/g; *P* = 0.001). Liver uptake was 9.3 ± 0.7%ID/g at 2 d p.i., which was slightly, but significantly lower than at 1 d p.i. (11.7 ± 2.7% ID/g; *P* = 0.02).

In NOD/SCID mice injected with [^111^In]In-DOTA-trastuzumab Fab, activity in the blood decreased from 9.3 ± 2.2% ID/g at 2 h p.i. to 3.1 ± 0.4% ID/g, 0.4 ± 0.07% ID/g and 0.2 ± 0.01% ID/g at 6 h, 18 and 24 h p.i., respectively (Figure 3C). Tumor uptake was 1.5 ± 0.2%ID/g, 1.9 ± 0.6% ID/g, 3.1 ± 0.5% ID/g and 2.6 ± 1.2% ID/g at 2, 6, 18 and 24 h p.i., respectively. Tumor uptake at 24 h p.i. was 4.0-fold significantly lower than [^111^In]In-DOTA-trastuzumab IgG at 2 d p.i. (10.6 ± 0.6% ID/g; *P* < 0.001). The kidneys exhibited the highest normal tissue uptake with 105.4 ± 9.5% ID/g at 2 h p.i. increasing to 118.5 ± 0.5% ID/g at 6 h p.i., then decreasing to 87.9 ± 11.1% ID/g at 24 h p.i. Spleen uptake was 2.3 ± 0.8% ID/g at 2 h but was not changed up to 24 h p.i. At 24 h p.i. spleen uptake was 13.2-fold significantly lower than [^111^In]In-DOTA-trastuzumab IgG at 2 d p.i. (29.0 ± 7.4% ID/g; *P* = 0.0012). Liver uptake at 24 h p.i. was 5.7 ± 0.7% ID/g.

**Figure 4:**
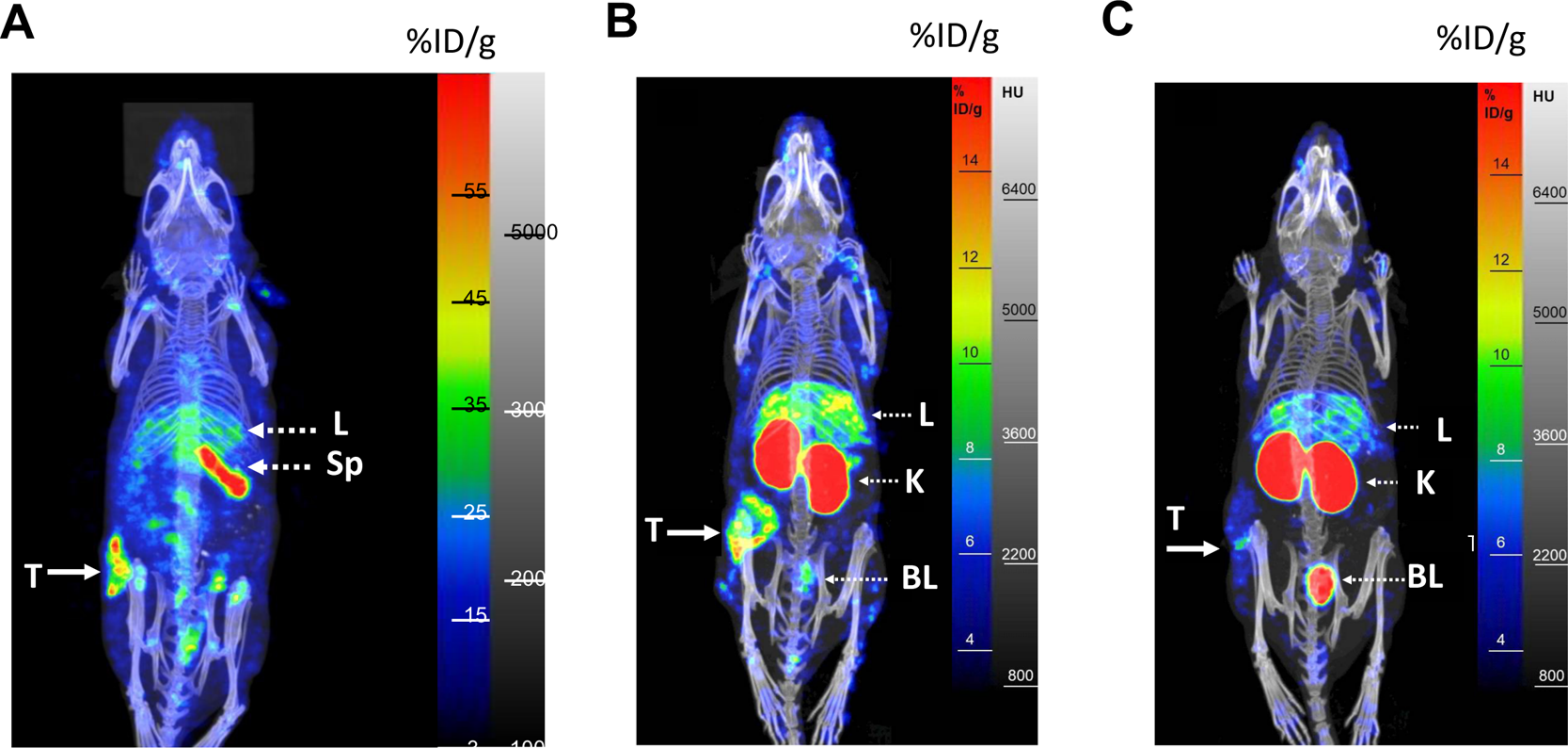
Representative SPECT/CT images of NRG mice (panel A) or NOD/SCID mice (panels B and C) with s.c. 164/8-1B/H2N.luc^+^ tumors injected with (A) [^111^In]In/[^225^Ac]Ac-DOTA-trastuzumab IgG at 2 d p.i., (B) [^111^In]In/[^225^Ac]Ac-DOTA-trastuzumab F(ab')_2_ at 2 d p.i., or (C) [^111^In]In/[^225^Ac]Ac-DOTA-trastuzumab Fab at 18 h p.i. T: tumor, L: liver, Sp: spleen, K: kidneys, BL: bladder. The intensity scale for the SPECT/CT images shows the percent injected dose/g (% ID/g). The CT intensity scale is shown in Hounsfield Units (HU). The image shown in panel A is reproduced from ref (19). Available under a CC-BY 4.0. Copyright 2023, Misaki Kondo, Zhongli Cai, Nubaira Forkin, Raymond M. Reilly.

SPECT/CT imaged s.c. 164/8-1B/H2N.luc^+^ tumors in mice at 2 d p.i. of [^111^In]In/[^225^Ac]Ac-DOTA-trastuzumab IgG (Figure 4A) or [^111^In]In/[^225^Ac]Ac-DOTA-trastuzumab F(ab')_2_ (Figure 4B) but tumors were not well-visualized at 18 h p.i. of [^111^In]In/[^225^Ac]Ac-DOTA-trastuzumab Fab (Figure 4C). Liver and especially spleen uptake were seen on images with [^111^In]In/[^225^Ac]Ac-DOTA-trastuzumab IgG, while the kidneys were most prominent for F(ab')_2_ or Fab. Liver uptake appeared higher for [^111^In]In/[^225^Ac]Ac-DOTA-trastuzumab F(ab')_2_ than Fab. Bladder activity was observed in mice receiving [^111^In]In/[^225^Ac]Ac-DOTA-trastuzumab F(ab')_2_ or Fab.

### Tumor and Normal Organ Dosimetry

The radiation absorbed doses in the tumor and normal organs for [^225^Ac]Ac-DOTA-trastuzumab IgG, F(ab')_2_ or Fab in NRG or NOD SCID mice with s.c. 164/8-1B/H2N.luc^+^ human BC xenografts are shown in Table 1. The tumor dose for [^225^Ac]Ac-DOTA-trastuzumab IgG, F(ab')_2_ or Fab was 0.22 ± 0.02 Gy/kBq, 0.48 ± 0.02 Gy/kBq and 0.14 ± 0.03 Gy/kBq, respectively, corresponding to 1.32 ± 0.12 Gy, 2.88 ± 0.12 Gy and 0.84 ± 0.18 Gy, respectively for an injected total amount of 6 kBq used for RIT studies. The highest normal organ dose for [^225^Ac]Ac-DOTA-trastuzumab IgG was in the spleen (2.9 ± 0.4 Gy/kBq), corresponding to 17.4 ± 2.4 Gy for 6 kBq. The spleen dose was greatly reduced in mice injected with [^225^Ac]Ac-DOTA-trastuzumab F(ab')_2_ (0.56 ± 0.12 Gy/kBq) which corresponded to 3.36Gy ± 0.72 Gy for 6 kBq. The spleen dose for [^225^Ac]Ac-DOTA-trastuzumab Fab was 0.018 ± 0.003 Gy/kBq, corresponding to 0.11 ± 0.02 Gy for 6 kBq. Kidney doses were higher for mice injected with [^225^Ac]Ac-DOTA-trastuzumab F(ab')_2_ or Fab than IgG. Doses to the kidneys were 0.11 ± 0.05 Gy/kBq, 0.82 ± 0.04 Gy/kBq and 1.32 ± 0.08 Gy/kBq for [^225^Ac]Ac-DOTA-trastuzumab IgG, F(ab')_2_ or Fab, respectively, corresponding to 0.66 ± 0.30 Gy, 4.92 ± 0.24 Gy and 7.92 ± 0.48 Gy, respectively for 6 kBq. The dose in the liver for [^225^Ac]Ac-DOTA-trastuzumab IgG, F(ab')_2_ or Fab was 0.26 ± 0.02 Gy/kBq, 0.18 ± 0.01 Gy/kBq and 0.12 ± 0.01 Gy/kBq, corresponding to 1.56 ± 0.12 Gy, 1.08 ± 0.06 Gy and 0.72 ± 0.06 Gy for 6 kBq.

**Table 1:** Absorbed Doses in the Tumor and Normal Organs in NRG or NOD SCID Mice with s.c. HER2-Positive 164/8-1B/H2N.luc^+^ Human Breast Cancer Xenografts Injected i.v. with [^225^Ac]Ac-DOTA-Trastuzumab IgG, F(ab')_2_ or Fab^a^

	absorbed dose (Gy/kBq)		
organ	[^225^Ac]Ac-DOTA-trastuzumab IgG^b^	[^225^Ac]Ac-DOTA-trastuzumab F(ab′)_2_^c^	[^225^Ac]Ac-DOTA-trastuzumab Fab^c^
blood	0.27 ± 0.03	0.08 ± 0.00	0.02 ± 0.00
brain	0.01 ± 0.00	0.00 ± 0.00	0.00 ± 0.00
heart	0.09 ± 0.01	0.05 ± 0.00	0.02 ± 0.00
lung	0.12 ± 0.04	0.05 ± 0.01	0.06 ± 0.03
kidney	0.11 ± 0.05	0.82 ± 0.04	1.32 ± 0.08
pancreas	0.03 ± 0.00	0.02 ± 0.00	0.01 ± 0.00
spleen	2.9 ± 0.4	0.56 ± 0.12^d^	0.02 ± 0.00
liver	0.26 ± 0.02	0.18 ± 0.01	0.12 ± 0.01
stomach	0.04 ± 0.01	0.02 ± 0.00	0.01 ± 0.00
intestine	0.11 ± 0.02	0.02 ± 0.00	0.01 ± 0.00
skin	0.04 ± 0.01	0.05 ± 0.01	0.04 ± 0.01
muscle	0.01 ± 0.00	0.01 ± 0.00	0.00 ± 0.00
bone	0.16 ± 0.02	0.02 ± 0.00	0.01 ± 0.00
tumour	0.22 ± 0.02	0.48 ± 0.02^d^	0.14 ± 0.03^d^
carcass	0.05 ± 0.00	0.04 ± 0.00	0.01 ± 0.00

a Doses were estimated as described in the Materials and methods based on biodistribution studies (Figure 3) in mice injected with [^111^In]In-DOTA-trastuzumab IgG, F(ab')_2_ or Fab coinjected with [^225^Ac]Ac-DOTA-trastuzumab IgG, F(ab')_2_ or Fab.

b Biodistribution studies in NRG mice.

c Biodistribution studies in NOD/SCID mice.

d The activity in these tissues reached a plateau and it was not feasible to derive an effective decay constant, thus (Ã_*t*–∞_) was estimated by assuming further elimination solely by physical decay.

### Normal Tissue Toxicity

**Figure 5:**
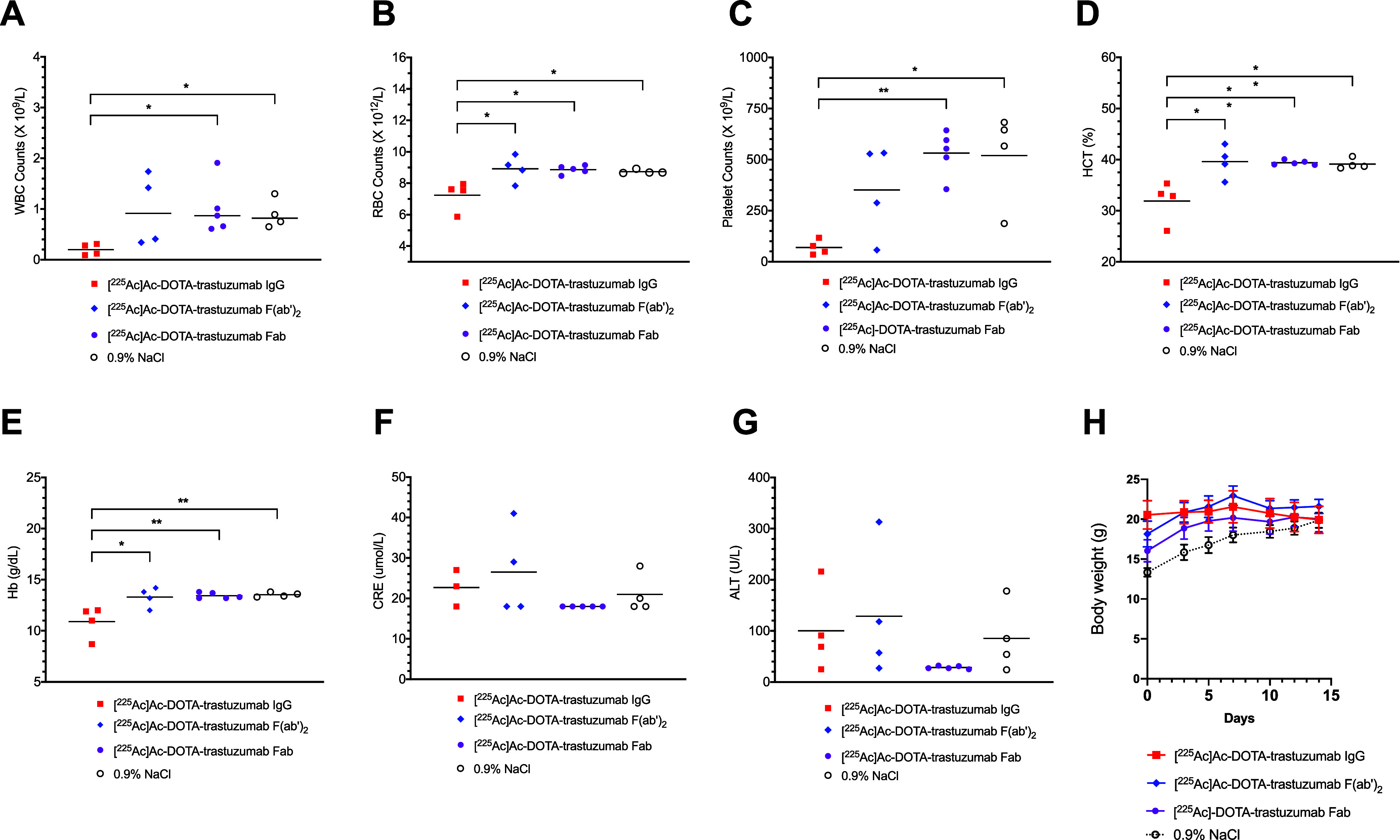
Hematology and blood biochemistry in NRG mice at 14 d p.i. of [^225^Ac]Ac-DOTA-trastuzumab IgG, [^225^Ac]Ac-DOTA-trastuzumab F(ab')_2_ , [^225^Ac]Ac-DOTA-trastuzumab Fab or 0.9% NaCl. (A) White blood cell (WBC) counts. (B) Red blood cell (RBC) counts. (C) Platelets (PLT). (D) Hematocrit (Hct). (E) Hemoglobin (Hb). (F) Creatinine (CRE). (G) Alanine aminotransferase (ALT). (H) Body weight. Data shown are values for individual mice and horizontal bars are the median value (*n* = 4–5). Significant differences (*P ≤ 0.05, **: *P* ≤ 0.01).

At 2 weeks p.i. of 6 kBq of [^225^Ac]Ac-DOTA-trastuzumab IgG administered as 2 kBq (40 μg) followed by 4 kBq (40 μg) separated by 8 d, NRG mice exhibited significantly decreased white blood cell (WBC) counts compared to [^225^Ac]Ac-DOTA-trastuzumab Fab (*P* = 0.024) or 0.9% NaCl (*P* = 0.011; Figure 5A). In contrast, WBC counts in mice injected with these amounts of [^225^Ac]Ac-DOTA-trastuzumab F(ab')_2_ were not decreased compared to 0.9% NaCl or [^225^Ac]Ac-DOTA-trastuzumab Fab. Red blood cell (RBC) counts were significantly lower for mice receiving [^225^Ac]Ac-DOTA-trastuzumab IgG than [^225^Ac]Ac-DOTA-trastuzumab F(ab')_2_ (*P* = 0.013) or Fab (*P* = 0.011) or 0.9% NaCl (*P* = 0.03; Figure 5B). Platelet (PLT) counts were significantly lower for mice injected with [^225^Ac]Ac-DOTA-trastuzumab IgG than Fab (*P* = 0.007) or 0.9% NaCl (*P* = 0.013) (Figure 5C). Hematocrit (HCT) and hemoglobin (Hb) were significantly lower for mice injected with [^225^Ac]Ac-DOTA-trastuzumab IgG than F(ab')_2_ (*P* = 0.005 and *P* = 0.013, respectively) or Fab (*P* = 0.004 and *P* = 0.006, respectively) or 0.9% NaCl (*P* = 0.008 and *P* = 0.007; Figure 5D,E). There were no significant differences in CRE or ALT in mice injected with [^225^Ac]Ac-DOTA-trastuzumab IgG, F(ab')_2_ or Fab compared to 0.9% NaCl (Figure 5F,G). There was no body weight loss in mice injected with any RIT agents (Figure 5H).

### Radioimmunotherapy

**Figure 6:**
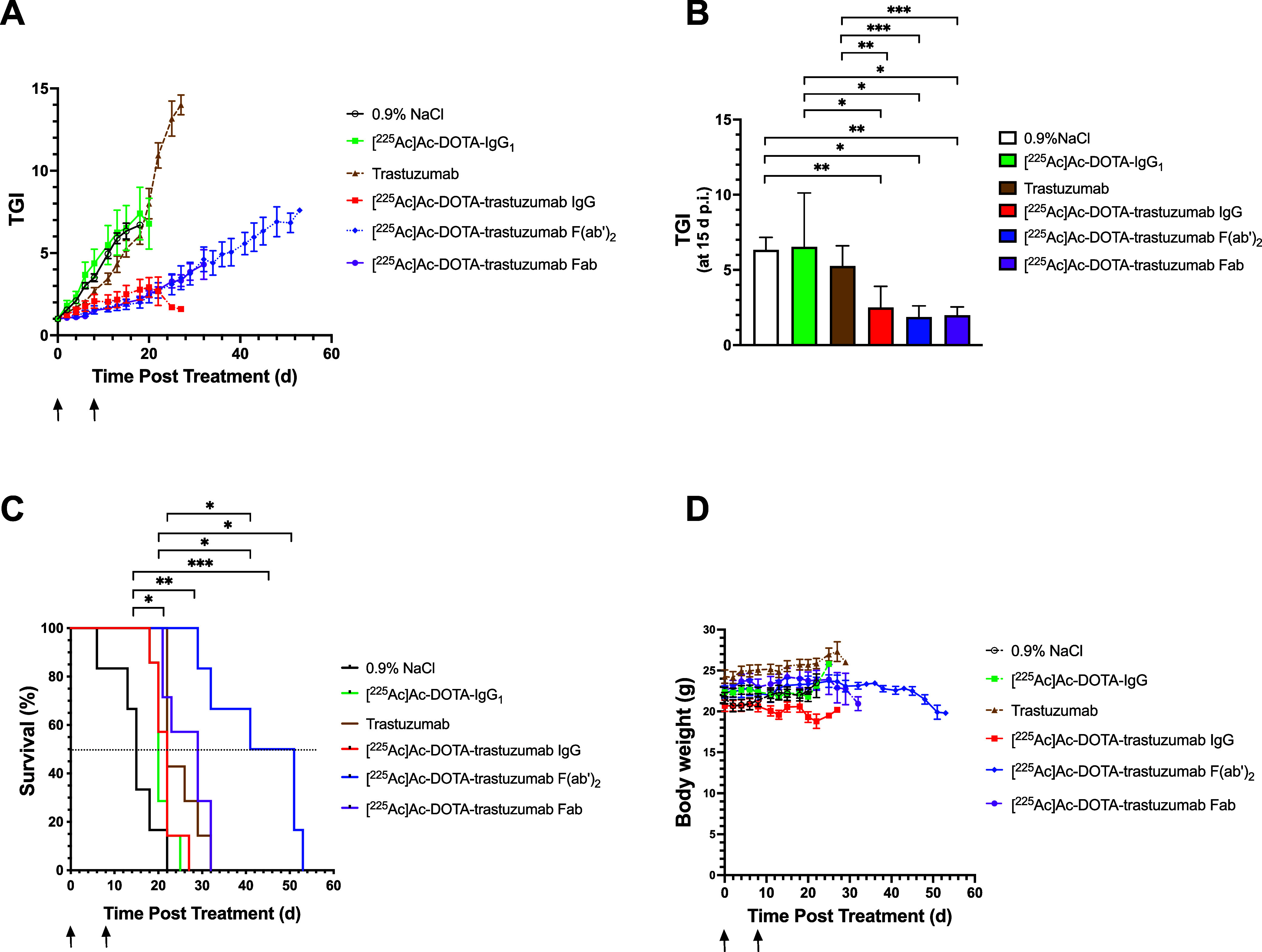
Treatment of NRG mice (*n* = 6–7) with s.c. 164/8-1B/H2N.luc^+^ tumors by i.v. injection of 2 kBq (40 μg) followed by 4 kBq (40 μg) separated by 8 d of irrelevant [^225^Ac]Ac-DOTA-IgG_1_ or [^225^Ac]Ac-DOTA-trastuzumab IgG, F(ab')_2_ or Fab (arrows). Control mice were treated with two doses of unlabeled trastuzumab (40 μg) or 0.9% NaCl. (A) Tumor growth index (TGI) vs time. (B) TGI at 15 d post-treatment (*n* ≥ 3 mice per group). (C) Kaplan–Meier survival. (D) Body weight. TGI and body weight values are mean ± SEM. Significant differences (*: *P* ≤ 0.05, **: *P* ≤ 0.01, ***: *P* ≤ 0.001) between TGI at 15 d. p.i. (panel B) or median survival (dotted line) compared to 0.9% NaCl, [^225^Ac]Ac-DOTA-IgG_1_ and unlabeled trastuzumab (panel B).

The TGI vs time (d) post-treatment of NRG mice with s.c. 64/8-1B/H2N.luc^+^ human BC tumors administered 6 kBq of [^225^Ac]Ac-DOTA-trastuzumab IgG, F(ab')_2_ or Fab as 2 kBq (40 μg) and 4 kBq (40 μg) separated by 8 d or in control mice treated with irrelevant [^225^Ac]Ac-DOTA-IgG_1_, trastuzumab IgG or 0.9% NaCl are shown in Figure 6A. Tumors grew rapidly in control mice compared to mice treated with [^225^Ac]Ac-DOTA-trastuzumab IgG, F(ab')_2_ and Fab. The TGI at 15 d post-treatment, when at least 3 mice remained alive in each group, are shown in Figure 6B. There were no significant differences in the mean TGI for mice treated with [^225^Ac]Ac-DOTA-IgG_1_ (6.5 ± 3.6) or trastuzumab IgG (5.6 ± 1.3; *P* > 0.05) compared to 0.9% NaCl (6.3 ± 0.8). In contrast, at 15 d post-treatment, the TGI for mice treated with [^225^Ac]Ac-DOTA-trastuzumab IgG, F(ab')_2_ or Fab was 2.5-fold (*P* = 0.0013), 3.4-fold (*P* = 0.002) and 3.2-fold (*P* = 0.0047) significantly lower, respectively, than mice injected with 0.9% NaCl. Compared to unlabeled trastuzumab IgG, [^225^Ac]Ac-DOTA-trastuzumab IgG, F(ab')_2_ or Fab decreased TGI at 15 d by 2.1-fold (*P* = 0.0028), 2.8-fold (*P* = 0.0002) and 2.6-fold (*P* = 0.0003), respectively. [^225^Ac]Ac-DOTA-trastuzumab IgG, F(ab')_2_ or Fab decreased the TGI at 15 d by 2.6-fold (*P* = 0.0251), 3.5-fold (*P* = 0.0133) and 3.3-fold (*P* = 0.0151), respectively, compared to [^225^Ac]Ac-DOTA-IgG_1_. There were no significant differences (*P* > 0.05) in TGI at 15 d between [^225^Ac]Ac-DOTA-trastuzumab IgG, F(ab')_2_ or Fab.

The survival of treated and control mice is shown in Figure 6C. Control mice receiving 0.9% NaCl, unlabeled trastuzumab IgG or irrelevant [^225^Ac]Ac-DOTA-IgG_1_ exhibited a median survival of 15 d, 22 d and 20 d, respectively. Compared to 0.9% NaCl, [^225^Ac]Ac-DOTA-trastuzumab IgG, F(ab')_2_ or Fab significantly increased median survival by 1.5-fold to 22 d (*P* = 0.013), 3.1-fold to 46 d (*P* = 0.0005) and 1.9-fold to 29 d (*P* = 0.0015), respectively. Compared to mice treated with [^225^Ac]Ac-DOTA-IgG_1_, [^225^Ac]Ac-DOTA-trastuzumab F(ab')_2_ or Fab significantly increased median survival by 2.1-fold (*P* = 0.0003) and 1.3-fold (*P* = 0.008), respectively. There were no differences in median survival between mice receiving [^225^Ac]Ac-DOTA-trastuzumab IgG or [^225^Ac]Ac-DOTA-IgG_1_ (*P* > 0.05). Only [^225^Ac]Ac-DOTA-trastuzumab F(ab')_2_ increased survival compared to unlabeled trastuzumab IgG (*P* = 0.0028). Comparing the three RIT agents, [^225^Ac]Ac-DOTA-trastuzumab F(ab')_2_ was significantly more effective at increasing median survival than IgG (*P* = 0.0005) or Fab (*P* = 0.0083). However, [^225^Ac]Ac-DOTA-trastuzumab Fab was more effective than [^225^Ac]Ac-DOTA-trastuzumab IgG at increasing survival (*P* = 0.0251). There was no major body weight loss (>20%) in treated or control groups of mice (Figure 6D).

## Discussion

We previously reported the construction and characterization of [^111^In]In- and [^225^Ac]Ac-DOTA-trastuzumab IgG as potential theranostic pairs for HER2-positive BC.^19^ In our earlier report, we showed that [^225^Ac]Ac-DOTA-trastuzumab IgG was highly cytotoxic in vitro to HER2-positive SK-BR-3 human BC cells by inflicting multiple lethal DNA double-strand breaks. We further estimated the cellular dosimetry of [^225^Ac]Ac-DOTA-trastuzumab IgG. However, in vivo RIT studies or in vivo dosimetry estimates were not performed. The current study was motivated by preliminary dose-finding experiments of [^225^Ac]Ac-DOTA-trastuzumab IgG in NRG mice in which we were unable to identify an administered amount that did not cause major normal tissue toxicity, particularly hematological toxicity. We hypothesized that the more rapid elimination and lower spleen uptake of [^225^Ac]Ac-DOTA-trastuzumab F(ab')_2_ and [^225^Ac]Ac-DOTA-trastuzumab Fab would make these RIT agents less hematologically toxic but their effectiveness for treating HER2-positive BC tumors in mice was not known, since their more rapid elimination could decrease tumor uptake, diminishing their effectiveness for RIT. The results of the current study revealed that RIT with [^225^Ac]Ac-DOTA-trastuzumab F(ab')_2_ and [^225^Ac]Ac-DOTA-trastuzumab Fab eliminated hematological toxicity (Figure 5) and further, these RICs significantly inhibited tumor growth in NRG mice with s.c. HER2-positive 164/8-1B/H2N.luc^+^ human BC tumors. (Figure 6A,B). However, [^225^Ac]Ac-DOTA-trastuzumab F(ab')_2_ was most effective for improving median survival (Figure 6C).

Trastuzumab F(ab')_2_ and Fab were produced by proteolysis of trastuzumab IgG, purified, and conjugated with 13.8 ± 1.3 DOTA/F(ab′)_2_ or 12.1 ± 0.5 DOTA/Fab, respectively (Figure 1) by reaction with NHS-DOTA. The number of DOTA conjugated per F(ab')_2_ or Fab were almost identical to DOTA conjugated per trastuzumab IgG (12.7 ± 1.2 DOTA) in our previous study.^19^ NHS-DOTA reacts with ε-amino groups on lysines and the N-terminal amines of trastuzumab. Trastuzumab IgG harbors 88 lysines including *K*^65^, an important lysine in the complementarity-determining region (CDR) of the heavy chains.^26^ We previously reported that the *K*_D_ for binding of [^111^In]In-DOTA-trastuzumab IgG to HER2-positive SK-BR-3 cells was 1.2 ± 0.3  × 10^–8^ mol/L.^19^ Here, the *K*_D_ of [^111^In]In-DOTA-trastuzumab F(ab')_2_ and Fab was increased to 1.2 ± 0.3 ×  10^–7^ and 1.4 ± 0.3  × 10^–7^ mol/L, respectively, i.e. 10-fold decreased HER2 binding affinity (Figure 2). There is a higher probability of DOTA conjugation to *K*^65^ in F(ab')_2_ and Fab than trastuzumab IgG, since *K*^65^ represents 2/52 lysines in F(ab')_2_ and 1/25–26 lysines in Fab, depending on whether papain cleaves IgG at aspartate or histidine residues on the heavy chain to produce Fab^26−28^ while *K*^65^ represents 2/88 lysines in trastuzumab IgG. Further, the monovalent nature of [^111^In]In-DOTA-trastuzumab Fab may decrease HER2 binding avidity. However, the *K*_D_-value of [^111^In]In-DOTA-trastuzumab Fab was almost identical to that of bivalent [^111^In]In-DOTA-trastuzumab F(ab')_2_. No other studies have reported the *K*_D_ of [^111^In]In-DOTA-trastuzumab F(ab')_2_. We previously determined that the K_D_ for binding of [^111^In]In-DOTA-trastuzumab Fab conjugated to a lower number of DOTA (3.7 ± 0.2 DOTA/Fab) to SK-BR-3 cells was 2.0 ± 0.02 × 10^–8^ mol//L,^29^ suggesting that the higher DOTA conjugation level in the current study may explain decreased HER2 binding affinity. Nonetheless, [^111^In]In-DOTA-trastuzumab F(ab')_2_ and Fab retained a high proportion of HER2-specific binding to SK-BR-3 cells, with 96.1 and 92.5% of binding competed by excess unlabeled trastuzumab IgG, respectively (Figure S2).

Biodistribution studies and SPECT/CT imaging were performed in NRG mice with s.c. 164/8-1B/H2N.luc^+^ tumors coinjected with [^111^In]In-DOTA-trastuzumab IgG, F(ab')_2_ or Fab and [^225^Ac]Ac-DOTA-trastuzumab IgG, F(ab')_2_ or Fab. For theranostic purposes in patients with HER2-positive BC, [^111^In]In-DOTA-trastuzumab IgG, F(ab')_2__2_ or Fab would be administered for SPECT/CT imaging prior to RIT, but our intent was to capture any potential cytotoxic effects of α-particle emissions of ^225^Ac that may decrease the tumor or organ weight and thus change the %ID/g values, since the biodistribution of the ^111^In-labeled RICs was used to estimate dosimetry of ^225^Ac-labeled RICs. The biodistribution of [^225^Ac]Ac-DOTA-trastuzumab IgG, F(ab')_2_ or Fab was not measured due to the very small amounts (4 kBq) administered to mice and potential inaccuracies in indirectly quantifying ^225^Ac based on measurement of γ-emitting ^221^Fr or ^213^Bi daughters in the absence of secular equilibrium, which may be the case if there is release and redistribution of these daughters after α-particle decay of ^225^Ac.^22^ The stability constant for the Ac^3+^-DOTA complex (*K*_ML_ = 19.5)^30^ is lower than In^3+^-DOTA (*K*_ML_ = 24.5),^31^ which could result in some differences in the biodistribution of the ^111^In- and ^225^Ac-labeled RICs. Further, release of recoil energy after α-particle decay, may disrupt the complexation of ^225^Ac by DOTA and ^225^Ac daughters may not remain bound by DOTA.^32^ Despite these challenges, similar biodistribution of intraperitoneally (i.p.) injected [^111^In]In-DOTA-trastuzumab IgG and [^225^Ac]Ac-DOTA-trastuzumab IgG was reported in mice with i.p. HER2-positive SK-OV-3 human ovarian cancer tumors.^21^

[^111^In]In-DOTA-trastuzumab IgG and F(ab')_2_ exhibited high tumor uptake of 10.6 ± 0.6% ID/g and 8.6 ± 1.5% ID/g, respectively at 2 d p.i. in mice with s.c. HER2-positive 164/8-1B/H2N.luc^+^ human BC xenografts (Figure 3). In contrast, the tumor uptake of [^111^In]In-DOTA-trastuzumab Fab was 3–4 fold lower than IgG or F(ab')_2_, although the *K*_D_ was similar to [^111^In]In-DOTA-trastuzumab F(ab')_2_ but 10-fold greater than IgG. Furthermore, tumors were imaged by SPECT/CT at 2 d p.i. of [^111^In]In-DOTA-trastuzumab IgG or F(ab')_2_ but were not well-visualized with [^111^In]In-DOTA-trastuzumab Fab (Figure 4). These results indicate that the elimination rate from the blood and extent of normal organ sequestration affect tumor uptake of these RICs. [^111^In]In-DOTA-trastuzumab Fab were more rapidly eliminated from the blood with 0.2 ± 0.01% ID/g at 24 h p.i. compared to 4.7 ± 0.1% ID/g for [^111^In]In-DOTA-trastuzumab F(ab')_2_ (Figure 3). Both [^111^In]In-DOTA-trastuzumab F(ab')_2_ and Fab exhibited high kidney uptake at 24 h p.i. (87.9 ± 11.1% ID/g and 70.2 ± 8.1% ID/g, respectively) but the kidney uptake of [^111^In]In-DOTA-trastuzumab F(ab')_2_ decreased 1.4-fold to 49.7 ± 5.5% ID/g at 2 d p.i. (Figure 3). Bladder activity was observed on SPECT/CT images of mice injected with [^111^In]In-DOTA-trastuzumab F(ab')_2_ and Fab (Figure 4). Kidney uptake of antibody fragments is well-known.^33^ High kidney uptake has been observed in patients injected with [^68^Ga]Ga-DOTA-trastuzumab F(ab')_2_ for PET imaging of HER2-positive BC.^34^ An unexpected finding was the rapid elimination of [^111^In]In-DOTA-trastuzumab IgG from the blood, which decreased threefold from 33.1 ± 4.3% ID/g at 1 h p.i. to 11.0 ± 2.7% ID/g at 2 d p.i. and to 0.06 ± 0.02% ID/g at 14 d p.i. (Figure 3). Rapid elimination was associated with high spleen uptake (29.0 ± 7.4% ID/g at 2 d p.i. which reached a maximum of 62.7 ± 11.5% ID/g at 7 d p.i.) (Figure 3). High spleen uptake was limited to [^111^In]In-DOTA-trastuzumab IgG, since the uptake of [^111^In]In-DOTA-trastuzumab F(ab')_2_ at 2 d p.i. was 3.8-fold significantly lower (*P* = 0.001) and uptake of [^111^In]In-DOTA-trastuzumab Fab was 13.7-fold significantly lower at 24 h p.i. (*P* = 0.0012). Spleen uptake of antibodies is mediated by interaction of the Fc-domain with Fcγ receptors on splenic macrophages.^35^ The high spleen uptake of [^111^In]In-DOTA-trastuzumab IgG may be explained by the low levels of circulating IgG in immunocompromised mice such as NRG mice which are B-cell deficient, resulting in avid interactions with Fcγ-receptors.^36^ Sharma et al.^36^ similarly reported high spleen uptake of ^89^Zr-labeled RICs in NOD-SCID mice, and NOD-SCID-gamma (NSG) mice that are B-cell deficient, but not in athymic (nude) mice that retain B-cells but are T-cell deficient. High spleen uptake of [^111^In]In-DOTA-trastuzumab IgG observed in NRG mice may not be observed in patients with HER2-positive BC due to functional B-cells and normal levels of circulating IgG. SPECT images in patients with HER2-positive BC injected with [^111^In]In-DTPA-trastuzumab IgG^37^ or PET with [^68^Ga]Ga-DOTA-trastuzumab F(ab')_2_ did not show high spleen uptake.^34^

Based on the biodistribution of [^111^In]In-DOTA-trastuzumab IgG, F(ab')_2_ and Fab (Figure 3), we estimated the radiation absorbed doses in the 164/8-1B/H2N.luc^+^ tumor and normal organs for [^225^Ac]Ac-DOTA-trastuzumab IgG, F(ab')_2_ and Fab, assuming that substitution of ^225^Ac for ^111^In would not change the biodistribution of these RICs (Table 1). A limitation of ^111^In as a surrogate for ^225^Ac for estimating absorbed doses is its shorter physical half-life (*t*_1/2p_ = 2.8 d vs 10 d, respectively) which may not completely capture the elimination phase of ^225^Ac activity in biodistribution studies. However, the effective half-life (*t*_1/2e_) which incorporates physical decay and biological elimination is the most important for designing biodistribution studies to estimate dosimetry.^23^ In our study, we employed four time points which were selected based on the relative elimination rates of [^225^Ac]Ac-DOTA-trastuzumab IgG, F(ab')_2_ or Fab and we fitted the elimination curves for all tissues except the tumor and spleen to a one-phase exponential decay curve to accurately capture the elimination phase of activity from these tissues. For the tumor and spleen, it was assumed that activity at the final measured time point would be further eliminated solely by radioactive decay, which may overestimate the retention of activity in these tissues. Another limitation of ^111^In is that it is a different element than ^225^Ac. Recently, ^226^Ac (*t*_1/2p_ = 29.4 h) which emits γ-photons [Eγ = 158 keV (17.5%) and 238 keV (26.9%)] that enable SPECT imaging and biodistribution studies has been proposed as a surrogate for ^225^Ac.^38^ However, the *t*_1/2p_ of ^226^Ac is much shorter than ^111^In and ^226^Ac emits four α-particles in its decay to ^210^Pb, thus it is not a purely theranostic radionuclide for pairing with ^225^Ac. [^225^Ac]Ac-DOTA-trastuzumab F(ab')_2_ provided the highest absorbed dose in the tumor corresponding to 2.9 Gy for a total fractionated amount of 6 kBq used for RIT studies, while the dose was 2.2-fold lower for IgG (1.3 Gy) and 3.4-fold lower for Fab (0.8 Gy; Table 1). The lower tumor dose for [^225^Ac]Ac-DOTA-trastuzumab IgG vs F(ab')_2_ and Fab was due to a smaller time-integrated activity (Ã_0–∞_) in the tumor. The highest normal organ dose for [^225^Ac]Ac-DOTA-trastuzumab IgG was in the spleen (17.4 Gy for 6 kBq) but this was decreased 5.1-fold for F(ab')_2_ (3.4 Gy) and 158-fold for Fab (0.11 Gy). Irradiation of the spleen by external beam radiotherapy (EBRT) is associated with lymphopenia in patients at doses >18 Gy.^39^ A spleen dose of 15 Gy in patients with neuroendocrine tumors treated with [^177^Lu]Lu-DOTATATE resulted in hematological toxicity.^40^ The doses in the kidneys for [^225^Ac]Ac-DOTA-trastuzumab F(ab')_2_ (4.9 Gy) and Fab (7.9 Gy) were 7.4-fold and 12-fold higher, respectively, than IgG (0.7 Gy) but these remained within the 23 Gy recommended upper limit for EBRT^41^ and the 40 Gy limit for peptide-targeted radiotherapy.^42^ The liver dose was 1.6 Gy, 1.1 and 0.7 Gy for [^225^Ac]Ac-DOTA-trastuzumab IgG, F(ab')_2_ and Fab, respectively (Table 1). These doses are within the tolerable range (28–32 Gy) for EBRT.^43^

[^225^Ac]Ac-DOTA-trastuzumab IgG (6 kBq) administered as 2 kBq and 4 kBq separated by 8 d caused hematological toxicity in NRG mice, as evidenced by decreased WBC, RBC, PLT, and Hb at 2 weeks post-treatment (Figure 5). This toxicity may be due to the high spleen uptake of [^225^Ac]Ac-DOTA-trastuzumab IgG, since no toxicity was found for [^225^Ac]Ac-DOTA-trastuzumab F(ab')_2_ or Fab, which exhibited much lower spleen uptake (Figure 3). As discussed, spleen irradiation has been associated with hematologic toxicity in patients receiving EBRT^39^ or peptide-targeted radiotherapy.^40^ There was no increase in ALT or CRE in mice receiving [^225^Ac]Ac-DOTA-trastuzumab IgG, F(ab')_2_ or Fab, indicating no liver or kidney toxicity, respectively. The absence of liver or kidney toxicity agrees with the tolerable doses in these organs (Table 1). However, it remains possible that toxicity to the liver or kidneys could be manifested at later times.^44,45^ Jaggi et al. reported histological damage to the kidneys in mice at >10 weeks post-treatment with ^225^Ac-labeled HuM195 antibodies.^46^ Body weight was not decreased in mice receiving any RIT agent indicating no general toxicity. It was not possible in our study to assess HER2-mediated normal tissue toxicity since trastuzumab does not bind to the murine c-erbB2/neu homologue of HER2.^47^ A transgenic mouse model that spontaneously develops a HER2-positive mammary carcinoma tumor and in which there is HER2 expression by normal tissues is useful for studying the effectiveness and toxicity of trastuzumab-based therapies.^48−50^ HER2-mediated tumor uptake of [^111^In]In-DTPA-trastuzumab IgG has been shown in these mouse models.^51^ However, a practical limitation is a long latency period for tumor development requiring 30–48 weeks in one study.^48^

Treatment of NRG mice with s.c. 164/8-1B/H2N.luc + human BC tumors with [^225^Ac]Ac-DOTA-trastuzumab IgG, F(ab')_2_ or Fab caused significantly greater tumor growth inhibition compared to unlabeled trastuzumab IgG, irrelevant [^225^Ac]Ac-DOTA-IgG_1_ or 0.9% NaCl (Figure 6A,B). However, RIT with [^225^Ac]Ac-DOTA-trastuzumab F(ab')_2_ was the most effective for increasing median survival (Figure 6C). The lower effectiveness of [^225^Ac]Ac-DOTA-trastuzumab IgG and Fab for increasing survival was due to mice reaching a humane end point requiring sacrifice due to toxicity or the tumor volume exceeding the maximum permitted size under the animal care protocol (<15 mm diameter). The absorbed dose in the tumor for [^225^Ac]Ac-DOTA-trastuzumab F(ab')_2_ was relatively modest (2.9 Gy), but α-particles have high relative biological effectiveness (RBE = 5)^52^ that may provide greater efficacy than expected based on the absorbed dose. Comparable results were obtained by Howe et al.^14^ for treatment of s.c. HER2-positive BT-474 tumors in athymic mice administered a single higher amount (9.2 kBq) of [^225^Ac]Ac-DOTA-trastuzumab IgG, in which tumor growth was inhibited but not arrested, despite the tumor dose (6.7 Gy) being 2.3-fold higher than in our study. Since tumor growth was not arrested and mice were not cured by administering a total of 6 kBq of [^225^Ac]Ac-DOTA-trastuzumab F(ab')_2_ but there was no major normal tissue toxicity (Figure 5), future studies could include additional fractionated amounts to improve response and survival.

## Conclusions

We conclude that [^111^In]In-DOTA-trastuzumab F(ab')_2_ and [^225^Ac]Ac-DOTA-trastuzumab F(ab')_2_ exhibited superior properties as a theranostic pair for SPECT/CT imaging and α-particle RIT of HER2-positive 164/8-1B/H2N.luc^+^ human BC tumors in NRG mice compared to [^111^In]In-DOTA-trastuzumab IgG and [^225^Ac]Ac-DOTA-trastuzumab IgG or [^111^In]In-DOTA-trastuzumab Fab and [^225^Ac]Ac-DOTA-trastuzumab Fab. [^111^In]In-DOTA-trastuzumab IgG or F(ab')_2_ imaged tumors by SPECT/CT but there was high spleen uptake of [^111^In]In-DOTA-trastuzumab IgG and tumors were not well-visualized with [^111^In]In-DOTA-trastuzumab Fab. The radiation absorbed dose estimated for the spleen for [^225^Ac]Ac-DOTA-trastuzumab F(ab')_2_ was much lower than [^225^Ac]Ac-DOTA-trastuzumab IgG, and doses in the kidneys were lower than [^225^Ac]Ac-DOTA-trastuzumab Fab. [^225^Ac]Ac-DOTA-trastuzumab IgG caused hematological toxicity, while no major normal tissue toxicity was observed for [^225^Ac]Ac-DOTA-trastuzumab F(ab')_2_ or Fab. Although all RICs inhibited tumor growth, [^225^Ac]Ac-DOTA-trastuzumab F(ab')_2_ was the most effective for increasing the median survival of NRG mice with 164/8-1B/H2N.luc^+^ tumors.

## Supporting Information Available

Methods of optimization of trastuzumab digestion to generate trastuzumab Fab. Methods of optimization of conditions for DOTA conjugation of trastuzumab F(ab')_2_ and Fab. Methods of assessing HER2 immunoreactivity of [^111^In]In-DOTA-trastuzumab IgG, F(ab')_2_ or Fab by single concentration binding assay. Methods of assessing subcellular distribution of [^111^In]In-DOTA-trastuzumab IgG and [^225^Ac]Ac-DOTA-trastuzumab IgG in SK-BR-3 cells by cell fractionation. Results of Western blot analysis of trastuzumab Fab generated by digestion of trastuzumab at varying papain digestion conditions. Results of single concentration binding assay for [^111^In]In-DOTA-trastuzumab IgG, F(ab')_2_ or Fab. Results of subcellular distribution of [^111^In]In-DOTA-trastuzumab IgG and [^225^Ac]Ac-DOTA-trastuzumab IgG in SK-BR-3 cells (PDF)
