# Supplementary material for: Preclinical Comparison of [111In]In- and [225Ac]Ac-DOTA-Trastuzumab IgG, F(ab′)2 and Fab for Theranostic SPECT/CT Imaging and α-Particle Radioimmunotherapy of HER2-Positive Human Breast Cancer
Source: Mol Pharm. 2024 Dec 12;22(1):474–87. doi: 10.1021/acs.molpharmaceut.4c01071 (PMC11708818; doi:10.1021/acs.molpharmaceut.4c01071)
Supplement: Supplementary file 1 — mp4c01071_si_001.pdf [file mp4c01071_si_001.pdf]

Preclinical Comparison of [^{111}In]In- and [^{225}Ac]Ac-DOTA-Trastuzumab IgG, F(ab′)$_2$ and Fab for Theranostic SPECT/CT Imaging and α-Particle Radioimmunotherapy of HER2-Positive Human Breast Cancer

Misaki Kondo [a], Zhongli Cai [a], Conrad Chan [a], Madeline K. Brown [a], Raymond M. Reilly [a,b,c,d*]

[a] *Department of Pharmaceutical Sciences, University of Toronto, Toronto, ON, M5S 3M2, Canada*

[b] *Department of Pharmaceutical Sciences, Leslie Dan Faculty of Pharmacy, University of Toronto, 144 College St., Toronto, ON M5S 3M2, Canada*

[c] *Department of Medical Imaging, Temerty Faculty of Medicine, University of Toronto, 263 McCaul St 4th floor, Toronto, ON M5S 1A8, Canada*

[d] *Joint Department of Medical Imaging and Princess Margaret Cancer Centre, University Health Network, 610 University Ave, Toronto, Ontario, M5G 2C1, Canada*

* Email: raymond.reilly@utoronto.ca; Tel. +1 416-946-5522

Supporting Information

MATERIALS AND METHODS

Optimization of conditions to generate trastuzumab Fab. To optimize the conditions for trastuzumab proteolysis by papain, trastuzumab IgG was reacted with papain at varying concentrations of 1 mg of papain resin (8 mL slurry) per 20 mg (1:20 w/w enzyme:IgG ratio), 50 mg (1:50 w/w enzyme:IgG ratio) and 100 mg (1:100 w/w enzyme:IgG ratio) of IgG as described in the main manuscript. Briefly, immobilized papain equilibrated with freshly produced 20 mM NaH_2PO_4 pH 7.0 buffer containing 10 mM Na_2EDTA and 80 mM L-cysteine (digestion buffer) was reacted with trastuzumab IgG buffer-exchanged in 20 mM NaH_2PO_4 buffer, pH 7.0 containing 10 mM Na_2EDTA in a 15 mL Conical Centrifuge tube (Fisher Scientific) containing nitrogen (N_2). The reaction was gently mixed for 20 h at 37 °C. After incubation, the supernatant of the resin suspension containing Fab was collected by repeated centrifugation (200 × g, 5 min). The generated Fab was filtered through a 0.22 μm filter to remove residual resin, and buffer-exchanged into 100 mM $NaHCO_3$ buffer, pH 8.2 by centrifugation (30 kDa MW cut-off). The Fab fragments generated under digestion conditions of 1:20, 1:50, and 1:100 w/w papain:IgG ratio were analyzed by Western blot to identify Fab and residual Fc. Briefly, 3 μg of trastuzumab Fab generated from trastuzumab IgG under varying papain concentrations were subjected to SDS-PAGE analysis on a 4-20% Mini-Protein Tris/Glycine mini-gel (BioRad). The protein bands were electrophoretically transferred onto an Immunoblot polyvinylidene fluoride (PVDF) membrane (BioRad) and probed with goat anti-human Fab or anti-human Fc specific) antibodies conjugated to HRP (Sigma-Aldrich, Product No. A0170 and A0293). Bands were visualized directly on the membrane by incubating with diaminobenzidine (DAB, Sigma-Aldrich) substrate and 0.03% H_2O_2.

Optimization of conditions for DOTA conjugation of trastuzumab F(ab′)$_2$ and Fab. To optimize the conditions for DOTA conjugation, trastuzumab F(ab′)$_2$ (15-20 mg/mL in 100 mM

$NaHCO_3$ buffer, pH 8.2) were reacted with a 20-fold, 30-fold, or 50-fold molar excess of NHS-DOTA for 2 h at room temperature (RT). Trastuzumab Fab (15-20 mg/mL in 100 mM $NaHCO_3$ buffer, pH 8.2) were reacted with a 40-fold or 60-fold molar excess of NHS-DOTA for 2 h at RT. DOTA-F(ab′)$_2$ were purified and buffer-exchanged into PBS, pH 7.4 by ultrafiltration on an Amicon device (MWCO=50 kDa) centrifuged at 7000 × g for 5 mins, repeated 5 times. DOTA-Fab was purified and exchanged into PBS pH 7.4 on an Amicon device (MWCO=30 kDa) centrifuged at 4800 × g for 5 mins, repeated 5 times. DOTA-F(ab′)$_2$ and DOTA-Fab were adjusted to 5 mg/mL and stored at 4 ºC. The number of DOTA per F(ab′)$_2$ or Fab was determined by matrix-assisted laser desorption ionization time-of-flight (MALDI-TOF) mass spectrometry (AIMS Mass Spectrometry Laboratory, Department of Chemistry, University of Toronto). The m/z value of the single-protonated mass spectral peak of DOTA-conjugated F(ab′)$_2$ or DOTA-Fab minus the m/z value for unconjugated fragments was divided by the mass of DOTA (387.41 Da) to estimate the number of DOTA per molecule. The target level of conjugation was 5-12 DOTA per F(ab′)$_2$ or Fab. The purity and homogeneity of DOTA-F(ab′)$_2$ and DOTA-Fab prepared under optimal conditions were determined by SDS-PAGE. Irrelevant DOTA-conjugated IgG$_1$, F(ab′)$_2$ or Fab were similarly prepared from non-specific human IgG$_1$ (Sigma-Aldrich Product No. 15154).

HER2 immunoreactivity assay. The HER2 immunoreactivity of [^{111}In]In-DOTA-trastuzumab IgG, F(ab′)$_2$ or Fab prepared by reaction with a 20-50-fold molar excess of NHS-DOTA was assessed in a single concentration binding assay using SK-BR-3 cells (4.2×10^5 HER2/cell) as reported.[1] Briefly, 1×10^6 SK-BR-3 cells in 1.5 mL Eppendorf tubes suspended in 100 μL of PBS, pH 7.4 were incubated with 10 nM (50 μL) of [^{111}In]In-DOTA-trastuzumab IgG, F(ab′)$_2$ or Fab for 3.5 h at 4°C. Binding was determined in the absence or presence of a 50-fold molar excess of trastuzumab IgG to measure total binding (TB) and non-specific binding (NSB), respectively. The

tubes were gently shaken every 30 min. After incubation, unbound activity was collected by centrifuging at 1,297 × g for 5 mins on an Eppendorf centrifuge 5424 (ThermoFisher Scientific, Waltham, MA), rinsing the cell pellet twice with 500 µL of ice-cold PBS and removing the supernatant. The cell pellets containing cell-bound activity (TB or NSB) were measured in a γ-counter. Specific binding (SB) was obtained by subtracting NSB from TB.

Subcellular fractionation assay. The subcellular distribution of [^{225}Ac]Ac-DOTA-trastuzumab IgG and [^{111}In]In-DOTA-trastuzumab IgG were compared in SK-BR-3 cells. Briefly, 1×10^5 cells were seeded into wells in a 24-well plate and cultured overnight. Cells were then incubated with 200 nmoles/L of [^{225}Ac]Ac-DOTA-trastuzumab IgG (3.7 kBq/µg) or [^{111}In]In-DOTA-trastuzumab IgG (0.22 MBq/µg) in 0.3 mL of growth medium for 16 h at 37 ˚C and 5% CO_2. The medium containing unbound RICs were removed, and the cells were rinsed twice with cold PBS. Cell surface-bound activity (CS) was collected by exposing the cells to 300µL of 0.2M Na acetate and 0.5M NaCl (pH 2.5) for 10 minutes on ice, then cells were rinsed twice with cold phosphate-buffered saline (PBS). Cells were then treated with 300µL of a commercial cell lysis buffer (Nuclei EZ lysis buffer; Sigma-Aldrich, St. Louis, MO) for 4–16 h at 4 °C. Cells were then detached by cell scraping, and the entire cell lysate was transferred to 1.5 mL Eppendorf tubes and centrifuged at 324 × g (Eppendorf Model 5424, Hamburg, Germany) for 5 min. The supernatant was collected which contained the cytoplasmic fraction. The cell pellets were collected, which contained the nuclei. The activities on the cell surface, in the cytoplasm, or in the nucleus were measured in a γ-counter, where the γ-emissions of ^{111}In or the ^{213}Bi daughter of ^{225}Ac were used to detect ^{225}Ac activity. The percentage of activity in each fraction was plotted as percent cell-bound activity.

RESULTS

Preparation and characterization of DOTA-trastuzumab F(ab′)$_2$ and Fab. Pure trastuzumab F(ab′)$_2$ or Fab were prepared by proteolytic digestion of trastuzumab IgG with immobilized pepsin or papain, respectively. The optimal enzyme:IgG ratio for preparing trastuzumab Fab were identified by Western blot probing for Fab or residual Fc. Enzyme:IgG ratios of 1:20, 1:50 or 1:100 all yielded trastuzumab Fab (**Fig. S1A**) but there was residual Fc at a 1:100 ratio, while a 1:20 or 1:50 ratio revealed no residual Fc (**Fig. S1B**). An enzyme:IgG ratio of 1:50 was selected for preparation of trastuzumab Fab. Reaction of trastuzumab F(ab′)$_2$ with a 20-fold, 30-fold or 50-fold molar excess of NHS-DOTA resulted in conjugation of 13.8 ± 1.3, 17.6 ± 2.3, and 21.8 ± 0.7 DOTA per molecule measured by MALDI-TOF analysis. Reaction of trastuzumab Fab with a 40-fold or 60-fold molar excess of NHS-DOTA resulted in conjugation of 12.1 ± 0.5, and 12.9 ± 1.2 DOTA per molecule, respectively. Trastuzumab IgG was reacted with a 30-fold molar excess of NHS-DOTA resulting in 12.7 ± 1.2 DOTA per molecule as previously reported [1].

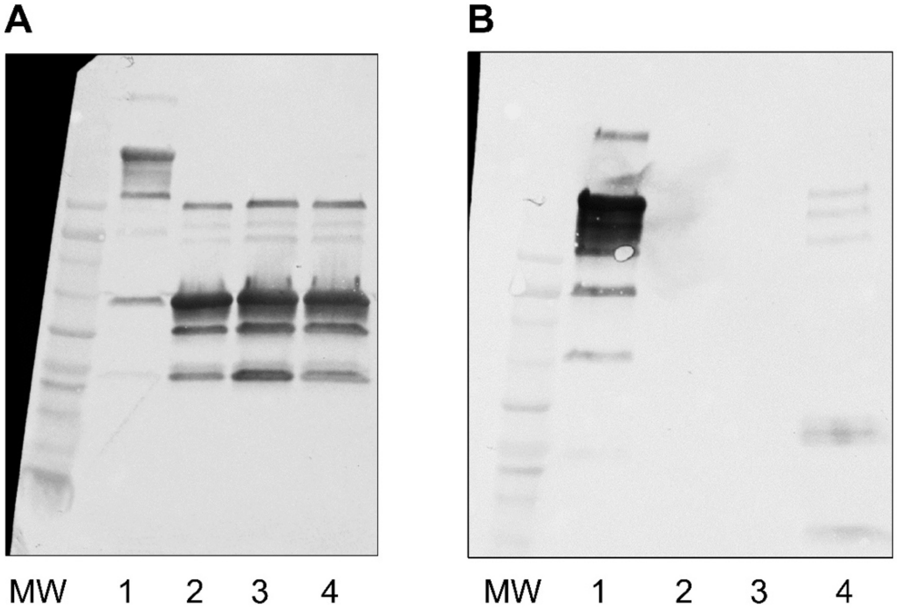

Figure. S1. Western blot analysis following SDS-PAGE under non-reducing conditions (see Fig. 1 in the main manuscript) of trastuzumab IgG (lane 1) and trastuzumab Fab digested with immobilized papain under a 1:20 (lane 2), 1:50 (lane 3) or 1:100 (lane 4) enzyme:IgG ratio, probed with goat anti-human IgG-horseradish peroxidase (HRP) immunoconjugates that are Fab specific (A) or Fc specific (B). Bands were detected by reaction with diamidobenzidine/0.03% H_2O_2.

Single concentration HER2 immunoreactivity assay. In a single concentration binding assay using HER2-positive SK-BR-3 cells, [^{111}In]In-DOTA-trastuzumab IgG exhibited the highest %SB at 98.2 ± 0.4% (**Figure S2A**). The %SB of [^{111}In]In-DOTA-trastuzumab F(ab′)$_2$ produced by reaction with a 20-fold, 30-fold, or 50-fold molar excess of NHS-DOTA were 96.2 ± 1.4%, 93.6 ± 1.6%, and 89.8 ± 0.6%, respectively (**Figure S2 B-D**). Only the 50-fold reaction significantly decreased the %SB compared to [^{111}In]In-DOTA-trastuzumab IgG (P=0.006). The %SB of [^{111}In]In-DOTA-trastuzumab Fab reacted with a 40-fold or 60-fold molar excess of NHS-DOTA were 92.5 ± 1.6% and 91.5 ± 0.6%, respectively (**Figure S2 E,F**). The %SB of [^{111}In]In-DOTA-trastuzumab Fab reacted with a 40-fold molar excess of NHS-DOTA was 92.5 ± 1.6% which was not significantly different than [^{111}In]In-DOTA-trastuzumab IgG (P=0.107; **Figure S2 E)**. The %SB of [^{111}In]In-DOTA-trastuzumab Fab reacted with a 60-fold molar excess of NHS-DOTA was 91.5 ± 0.6% which was significantly lower than [^{111}In]In-DOTA-trastuzumab IgG (**Figure S2 F**; P=0.0087). Based on these results, reaction of trastuzumab F(ab′)$_2$ with 20-fold molar excess of NHS-DOTA and reaction of trastuzumab Fab with 40-fold molar excess of NHS-DOTA were selected as the optimal DOTA conjugation conditions to produce DOTA-trastuzumab F(ab′)$_2$ and DOTA-trastuzumab Fab.

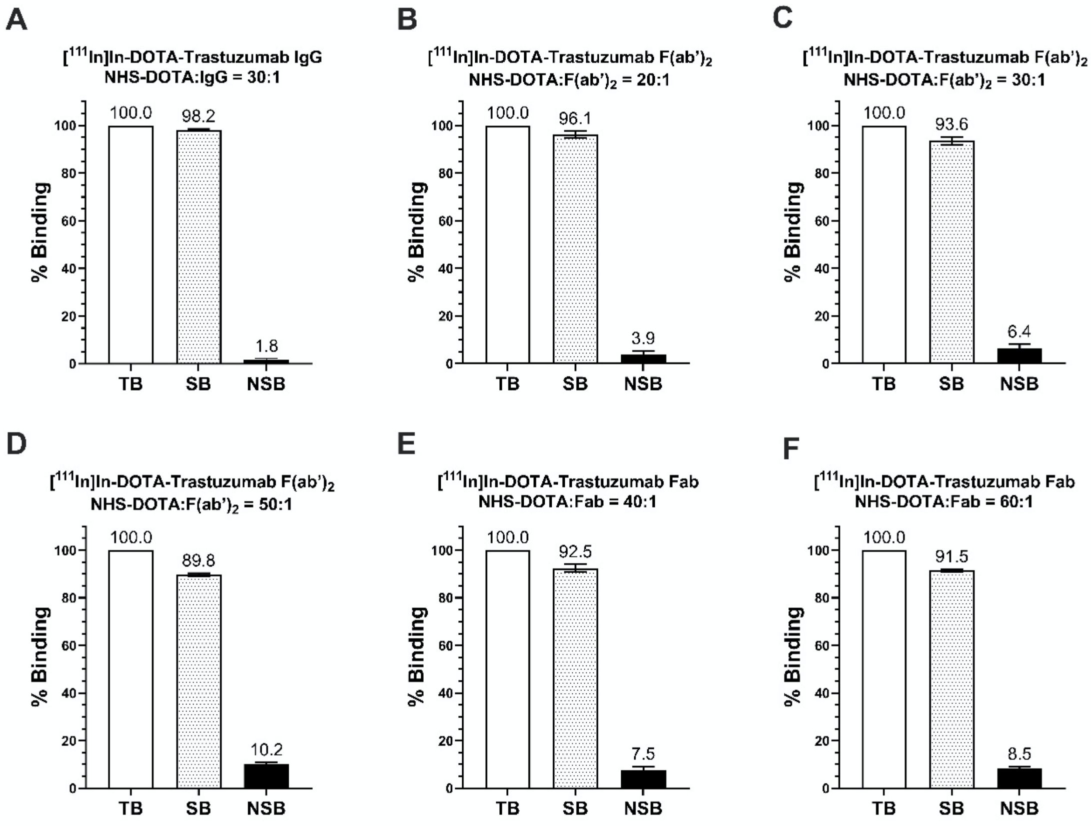

Figure S2. Single concentration binding assay results for (A) [^{111}In]In-DOTA-trastuzumab IgG produced at a 30:1 molar ratio of NHS-DOTA:IgG, (B-D) [^{111}In]In-DOTA-trastuzumab F(ab′)$_2$ produced at a 20:1, 30:1 or 50:1 molar ratio of NHS-DOTA:F(ab′)$_2$ and (E-F) [^{111}In]In-DOTA-trastuzumab Fab produced at a molar ratio of 40:1 or 60:1. Binding was determined in the absence (total binding; TB) or presence (non-specific binding; NSB) of a 50-fold molar excess of trastuzumab IgG. Specific binding (SB) was obtained by subtracting NSB from TB.

Subcellular fractionation assay. In a subcellular fractionation assay using HER2-positive SK-BR-3 cells, both [^{225}Ac]Ac-DOTA-trastuzumab IgG and [^{111}In]In-DOTA-trastuzumab IgG were found to have the highest cell-bound activity on the cell surface, followed by the cytoplasm, then

nucleus, when SK-BR-3 cells were treated with 200 nM of [^{225}Ac]Ac-DOTA-trastuzumab IgG (3.7 kBq/µg) or [^{111}In]In-DOTA-trastuzumab IgG (0.22 MBq/µg) for 16 h (**Figure S3**). There was no significant difference between the subcellular uptake of [^{225}Ac]Ac-DOTA-trastuzumab IgG and [^{111}In]In-DOTA-trastuzumab IgG for the cell surface, cytoplasm, or nucleus. On the cell surface, [^{225}Ac]Ac-DOTA-trastuzumab IgG demonstrated a slightly higher cell-bound activity of 74.2 ± 0.7% compared to [^{111}In]In-DOTA-trastuzumab IgG (62.7 ± 9.4%), but this difference was not significant (*P*=0.1669). In the cytoplasm, the percent cell-bound activity of [^{225}Ac]Ac-DOTA-trastuzumab IgG was 15.6 ± 0.4%, which was not significantly different than [^{111}In]In-DOTA-trastuzumab IgG (21.8 ± 4.8%; *P*=0.1507). Similarly, the percent cell-bound activity of [^{225}Ac]Ac-DOTA-trastuzumab IgG and [^{111}In]In-DOTA-trastuzumab IgG in the nucleus was 10.2 ± 0.3% and 15.2 ± 4.7%, respectively, which was not significantly different (*P*=0.1908).

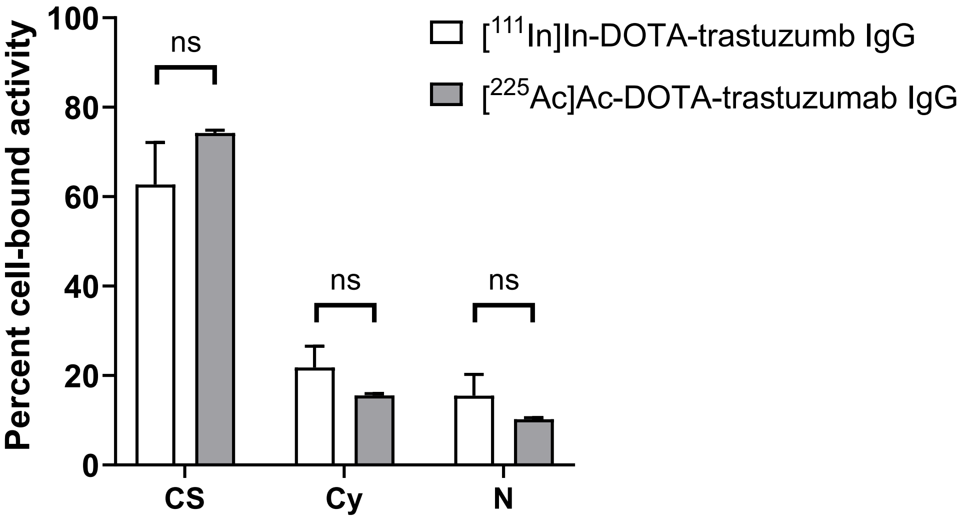

Figure S3. Subcellular distribution of activity (percent cell-bound ^{111}In or ^{225}Ac) for SK-BR-3 cells incubated with 200 nM of [^{225}Ac]Ac-DOTA-trastuzumab IgG or [^{111}In]In-DOTA-trastuzumab IgG

incubated for 16 h at 37 ˚C. Data shown are mean ± SD (n = 3). CS: cell surface. Cy: cytosol. N: nucleus. ns: not significant (*P*>0.05).

REFERENCES

(1) Kondo, M.; Cai, Z.; Chan, C.; Forkan, N.; Reilly, R. M. [^{225}Ac]Ac- and [^{111}In]In-DOTA-trastuzumab theranostic pair: cellular dosimetry and cytotoxicity in vitro and tumour and normal tissue uptake in vivo in NRG mice with HER2-positive human breast cancer xenografts. *EJNMMI Radiopharm Chem* **2023**, *8* (1), 24. DOI: 10.1186/s41181-023-00208-0.
